# Women’s Empowerment Metric for National Statistical Systems (WEMNS): Development and psychometric assessment of a face-to-face survey module

**DOI:** 10.1371/journal.pone.0345742

**Published:** 2026-05-08

**Authors:** Kathryn M. Yount, Agnes Quisumbing, Ruth Meinzen-Dick, Hazel Malapit, Md. Zahidul Hassan, Shelton S. E. Kanyanda, Sudhindra Sharma, Md. Imrul Hassan, Jessica Heckert, Flor Paz, Pankaj Pokhrel, Wilbert D. Vundru, Cheryl Doss, Greg Seymour, Heather Moylan, Talip Kilic, Emily Myers, Simone Faas, Yuk Fai Cheong, Sheela S. Sinharoy

**Affiliations:** 1 Rollins School of Public Health, Emory University, Atlanta, Georgia, United States of America; 2 International Food Policy Research Institute, Washington, District of Columbia, United States of America; 3 Data Analysis and Technical Assistance (DATA), Dhaka, Bangladesh; 4 National Statistical Office of Malawi, Zomba, Malawi; 5 Interdisciplinary Analysts, Kathmandu, Nepal; 6 World Bank, Washington, D.C., United States of America; 7 Department of Economics, Tufts University, Medford, Massachusetts, United States of America; 8 Department of Psychology, Emory University, Atlanta, Georgia, United States of America; South China Normal University, CHINA

## Abstract

Data to monitor progress towards gender-related Sustainable Development Goals (SDGs) remain limited. A comprehensive, concise set of metrics is needed for routine national data collection to monitor progress toward these goals. Our team developed and tested the Women’s Empowerment Metric for National Statistical Systems (WEMNS) survey module for use by national statistical offices and survey organizations in low- and middle-income countries (LMICs) to measure women’s and men’s empowerment. This paper summarizes the process of developing the WEMNS module and presents detailed results of a psychometric assessment of face-to-face surveys in Bangladesh, Malawi, and Nepal. Exploratory factor analysis and confirmatory factor analysis (CFA) in the pooled sample confirmed that most of the 13 item sets had adequately identified factor structures aligned with specific empowerment constructs and gender-related SDGs. In multi-group CFA assessing measurement equivalence of item sets across gender and countries, configural invariance was observed for 9 of 13 item sets across genders and across country settings. At least partial scalar invariance was observed for one item set across genders and no item sets across country settings. Spearman pairwise correlations among WEMNS factor scores derived from final CFA models showed weak associations, suggesting item sets were weakly related and distinct. Overall, Spearman pairwise correlations of 13 WEMNS-derived factor scores with external measures for basic needs, resources, agency, and subjective well-being were weak, but five moderately high correlations were conceptually aligned. In sum, the WEMNS measures require refinement and further psychometric assessment to confirm their use to make valid comparisons of empowerment across country settings and gender.

## Introduction

**Fig 1:**
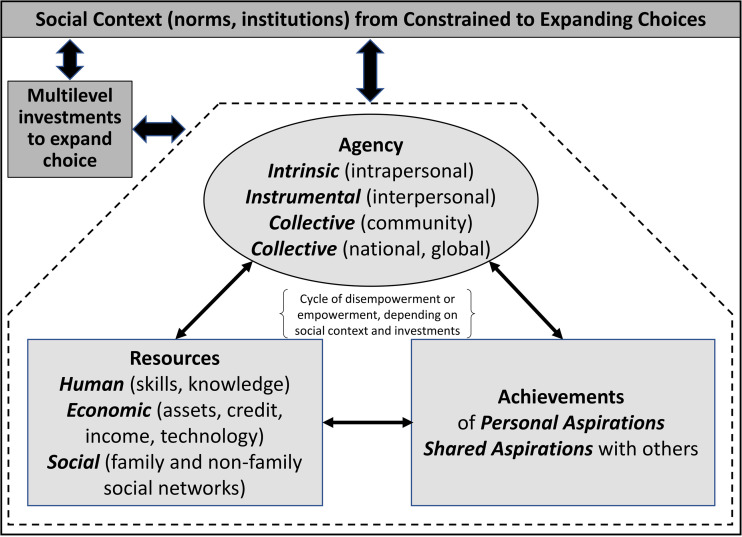
Women’s multidimensional, multilevel empowerment in social context.

*Women’s empowerment* is a *multidimensional* and *multilevel* concept that encompasses claims on new human, social, and economic resources. These claims potentiate transformations in individual, interpersonal, and collective agency, or the ability to imagine, act on, and realize personal and shared aspirations [1]. Thus, women’s empowerment is a process of actualization—of oneself and of collectives—that arises in an evolving structural and normative context characterized by historical constraints and expanding choices. The process of empowerment is multidirectional, involving reciprocal influences of investments in resources, changes in agency, and realizing aspirations over time (Fig 1).

Resource investments in women are central to global health and sustainable development. *Food-system interventions with women’s empowerment components* have had favorable effects on measures of food security, affordability, availability, diet quality and adequacy, and BMI for age [2]. Women’s engagement in *self-help groups*, an investment in social capital, has had positive effects on their economic empowerment, mobility, and political empowerment; mixed or null effects on their interpersonal agency (family-size decision making); and some effects on their individual agency (e.g., self-confidence and gender-equitable attitudes) [3,4]. While not ensuring positive outcomes, *economic transfers to women* have improved children’s well-being, health, and education [5], and *economic interventions with ‘gender-sensitivity’ social-norms components* may reduce partner violence [6,7] and increase women’s antenatal visits [8]. The effects of women’s empowerment interventions on multidimensional measures of their agency show promise [9,10]; however, synthesizing this evidence is limited by the heterogeneous conceptualization and measurement of women’s agency [11–13].

Approaches to measuring women’s empowerment have strengths and challenges. First, long-form measures of women’s empowerment capture more dimensions with more questions, potentially offering a fuller picture of women’s lives [14]. However, lengthy surveys increase respondent burden [15], potentially compromising data quality. Moreover, enumerators may lack the skills to clarify many nuanced questions on women’s empowerment, resulting in redundant responses to questions intended to capture different constructs [16]. These challenges may make long-form women’s empowerment surveys burdensome, costly, and of variable quality for routine data collection [15]. Second, abbreviated surveys of women’s empowerment have been sector-specific [17] or focused on selected dimensions of women’s empowerment [18], which provide an incomplete picture of women’s intersectoral, multifaceted lives [19]. Finally, women’s empowerment surveys often focus on sub-populations, such as adolescents [20] *or* women of reproductive age [21]. This approach produces measures of women’s empowerment tailored to specific periods of the life course but precludes tracking change in women’s empowerment *across* the life course and comparing age-specific and overall estimates of women’s empowerment for adolescent and adult populations across countries.

As a result, only 42% of the data needed to monitor Sustainable Development Goal (SDG) 5 and other gender-related SDGs is routinely produced [22]. At this pace, over two decades would be needed to fill gender-related data gaps. A concise generalized measure is needed for routine administration by national statistical offices (NSOs) and global survey platforms [23] that covers concepts of empowerment aligned with Sustainable Development Goal (SDG) 5 to “achieve gender equality and empower all women and girls,” and other gender-specific SDGs, such as SDG 1 (no poverty) and SDG 10 (reduced inequalities).

Relevant survey platforms include the Demographic and Health Surveys (DHS), United Nations Children’s Fund Multiple Indicator Cluster Surveys (UNICEF MICS), World Values Surveys (WVS), World Bank Living Standards Measurement Study (WB LSMS), World Bank Living Standards Measurement Study–Integrated Surveys on Agriculture (WB LSMS-ISA), and the 50x2030 Initiative to Close the Agricultural Data Gap (50x2030 Initiative) (Table 1). These platforms differ in their analytical objectives, and as such, their sampling designs and interview targets also differ. A concise empowerment module needs to consider these differences across survey platforms and the unique benefits of integrating a common module into each platform (Table 1).

**Table 1:** Benefits of integrating WEMNS into global survey platforms.

Survey	Overview	Scope	Sample	Data Priorities	Integrating WEMNS permits comparison of links between:
**DHS**	Repeated, X-sectional (RX) multi-topic national household survey (MTNHHS)	LMICs	Women 15–49 years; adult Men	•Population, health, nutrition	•WE (women’s empowerment)/ME (men’s empower-ment) with demographic/health outcomes
**UNICEF MICS**	RX-MTNHHS	LMICs	Women 15–49 years; Children 5–17 years	•Children’s and women’s health, health, education, child protection, HIV/AIDS, Water Sanitation and Hygiene, well-being •Data source for >30 SDG indicators	•WE with child/maternal outcomes
**WB-LSMS**	MTNHHS	LMICs	Households, Individuals	•Welfare, other key SES variables •Survey method innovations •NSO capacity strengthening	•WE with consumption, nutrition, non-monetary welfare
**WB-LSMS-ISA**	Longitudinal MTNHHS	LMICs	Households, Individuals	•Welfare, other SES variables •Survey method innovations •NSO capacity strengthening	•Longitudinal WE, ME processes with changes in: individual health, well-being, HH spending, community-level norms, institutional policy/reform, climate shocks
**WVS**	RX-MTNHHS	Income-diverse countries	Adults ≥18 years	•Human beliefs, values, links with social, political life • > 700 indicators	•Individual, community, national beliefs/values/ norms for changes in WE
**50x2030 Initiative**	Repeated, panel rotation agricultural household survey	LMICs	Adults ≥18 years	•Capacity strengthening •Data use •Research	•WE and agri-food sector outcomes

To meet this need, our global partnership developed the Women’s Empowerment Metric for National Statistical Systems (WEMNS) survey module. We worked with the 50x2030 Initiative to develop, test, and refine WEMNS as a concise, theoretically informed, psychometrically valid, and scalable module that NSOs and survey organizations in LMICs could adopt wholly or in part to measure women’s and men’s empowerment in national and sub-national household surveys. Users then could calculate specific WEMNS indicators and a counting-based WEMNS index [24,25]. Here, we report findings from Phase III of this project, contributing evidence on the feasibility, acceptability, and suitability of administering a concise, conceptually comprehensive face-to-face survey module to compare empowerment measures for adult women and men across three LMIC settings.

## Materials and methods

### Ethical considerations

The Institutional Review Board (IRB) of Emory University reviewed the study protocol for Nepal and determined that the research was exempt from further review and approval (STUDY00002762). The IFPRI IRB approved the research protocols for Bangladesh and Malawi (PHND-21–0518). Approval from a national entity was not required for non-biomedical studies in Bangladesh and Nepal, nor for work conducted by the NSO in Malawi. Eligible participants were informed in lay language about the study purpose, procedures, privacy of their responses, voluntariness of their participation and right to refuse any question and to withdraw at any time, foreseen risks of participation and safeguards against foreseen risks, and benefits of participation. Each eligible participant then was invited to ask any questions. The interviewer initiated the face-to-face survey with participants who provided verbal informed consent.

### Development of the WEMNS module

**Fig 2:**
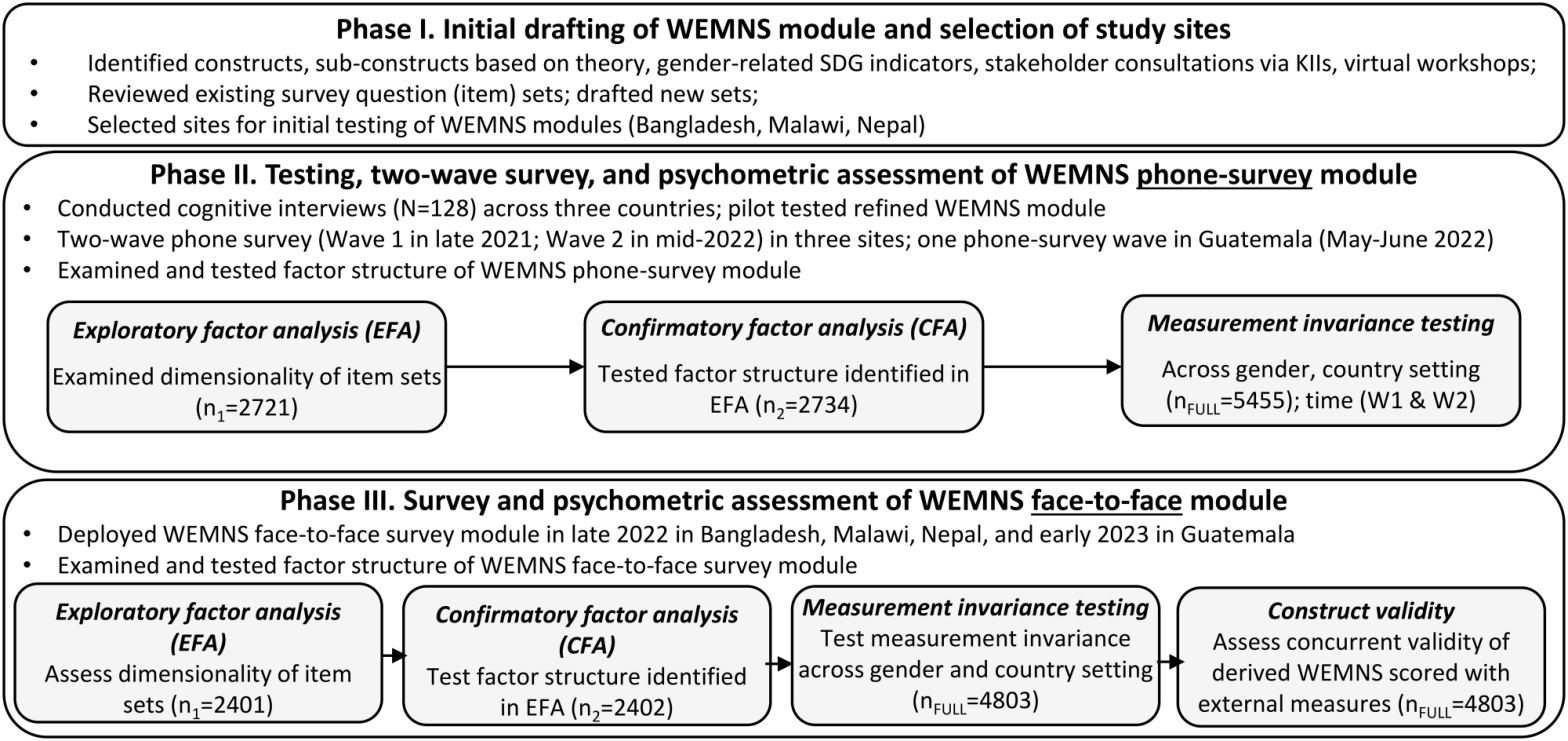
Three-phase approach to WEMNS module development and testing.

Development of the WEMNS module was implemented in three phases (Fig 2). Phases I and II are summarized in Fig 2 and are described briefly here, as these phases are described in detail elsewhere [26]. In Phase I, we identified theoretically relevant constructs and aligned them with gender-related SDG targets and indicators. We reviewed existing women’s empowerment questionnaires to operationalize identified constructs. We conducted key-informant interviews with 59 experts from governmental, civil-society, academic, and bilateral or multilateral organizations across Africa, Asia, and Latin America. Finally, we conducted two virtual, group-based stakeholder consultations [19]. All of these steps informed the development and refinement of an initial WEMNS module.

Phase II involved phone-based cognitive interviews (N = 128) and pilot surveys (N = 100 in Bangladesh, N = 82 in Malawi, N = 50 in Nepal) to refine further the initial WEMNS module. An intermediate WEMNS module then was implemented in a single phone survey in Guatemala (N = 2,031) and two-wave phone surveys in Bangladesh (N = 1807), Malawi (N = 1,657), and Nepal (N = 2,461). Psychometric assessment of data from the two-wave phone surveys (N = 5,455) revealed that WEMNS item sets captured common constructs of empowerment; however, few item sets achieved scalar invariance, indicating that the remaining item sets may not measure their underlying empowerment construct in equivalent ways across genders, country settings, or time. All of the steps undertaken in Phases I and II informed revisions to the WEMNS module that was implemented in Phase III. Phase III involved cross-sectional face-to-face surveys in the same country settings plus Guatemala and a psychometric assessment of those data. Guatemala was excluded from the psychometric assessment because it was not part of the original, three-country protocol, and was implemented later. Phase III is detailed here.

### Phase III. Survey and psychometric assessment of WEMNS module

#### Study sites and samples.

In all three country settings, a multi-stage probability sample design was implemented to identify households with at least one woman and one man 18–64 years. A target sample size of approximately 800 households per country was adequate for psychometric analysis [27] and indicator development [28]. Within each household, one eligible woman and one eligible man were randomly selected from the household roster. The sampling design differed across country settings because sampling frames differed (S1 File). These variations are of less concern in psychometric assessments since arriving at nationally representative estimates was not the analytical aim. Sample recruitment and data collection took place in 2022 from November 2 to November 27 in Bangladesh, from October 11 to November 17 in Malawi, and from December 4 to December 22 in Nepal.

#### Piloted WEMNS module.

Table 2 presents the dimensions in the face-to-face WEMNS module and psychometrically assessed here, number of items and response options that operationalize each dimension, and SDG targets/indicators and theoretical constructs of empowerment that each WEMNS dimension was designed to capture. These item sets are updated from those administered in the Phase II phone-survey [26] and align more closely with those selected for the Alkire-Foster-based index [24].

**Table 2:** WEMNS dimensions,^a^ Gender-related SDGs, and theoretical domains of empowerment.

SDG	Empowerment Domain	WEMNS Dimensions	# of items	Response Options	Example Item
**1**	Economic resources	Access to/ use of financial services^b^	8	Yes/No	In the past 12 months, have you ever: deposited or received money into or withdrawn money from any bank account, either yours or anyone else’s?
	Economic resources	Access to credit	5	Yes/No	Could you take a loan from any of the following: Bank or formal financial institution
**5.b**	Technological resources	Use of ICT^c^	6	1 = Daily; 5 = Never^d^	For each type of technology, please indicate whether you use the technology daily, weekly, monthly, less than monthly, or never.
**5.3**	Intrinsic economic agency	Endorsement of women’s freedom to choose livelihoods	5	0 = fully disagree; 3 = fully agree	Every woman should be free to choose whether to work for pay.
**5.6**	Intrinsic reproductive agency	Endorsement of women’s freedom to choose family formation	4	0 = fully disagree; 3 = fully agree	Every woman should be free to choose when to get married.
**10.3.1**	Intrinsic bodily agency	Rejection of women’s subjection to sexual harassment	5	1 = never acceptable; 3 = usually acceptable	A man prevents a woman from doing certain kinds of work, even if she wants to?
**5.4**	Intrinsic time-use agency	*Rejection of gender inequality in time spent on paid/unpaid activities* ^e^	4	0 = fully disagree; 3 = fully agree	Compared to a woman, a man can change his daily schedule more easily.
**5.4**	Instrumental time-use agency	Extent of influence over own time allocation	6	1 = no influence; 3 = a lot of influence	During the last 7 days, did you have no influence, some influence, or a lot of influence in decisions about the amount of time you spent on [ACTIVITY]?
**1/5.a**	Instrumental economic agency	Decision-making and control over income^f^	4	1 = no influence; 3 = a lot of influence	How much influence do you have regarding control over any source of income?
**5.5**	Collective agency in organizations	*Confidence to participate in community organizations* ^e^	5	1 = not at all confident; 3 = very confident	Are you not at all confident, somewhat confident, or very confident in your ability to participate in [ORGANIZATION] if you wanted to?
	Collective agency in organizations	Participation and leadership in community organizations^g^	5	0 = did not participate 1 = participated 2 = leader	In the last 12 months, have you participated in [ORGANIZATION]? In the last 12 months, have you acted in a leadership position in [ORGANIZATION]?
	Collective agency in communities	Perceptions of women’s community engagement^h^	9	0 = fully disagree; 3 = full agree	Women can really understand what is going on with your community
	Collective agency in communities	*Perceptions of men’s community engagement* ^e^	9	0 = fully disagree; 3 = full agree	Men can really understand what is going on with your community

^a^Three observed empowerment dimensions were included in the multidimensional WEMNS index, but not psychometrically assessed: legally documented ownership, secure tenure rights, secure transfer rights [24]; ^b^ The indicator in the multidimensional WEMNS index included 4 items (use of financial of services); ^c^ The indicator in the multidimensional WEMNS index included 2 items (mobile phone, internet), which are too few to model using psychometric methods; ^d^ Response options for the item set on use of ICT were dichotomized to Ever/ Never Used for the multi-group confirmatory factor analysis by country. ^e^ Item sets in italics were not included in the multidimensional WEMNS index; ^f^ The multidimensional WEMNS index separated this into 2 indicators (influence in spending decisions; influence in own health decisions) based on 3 items. The psychometric analysis included decisions over minor purchases; ^g^ The multidimensional WEMNS index separated this into 2 indicators (participation in orgs; leadership in orgs); ^h^ The multidimensional WEMNS index included 4 items.

The WEMNS module for face-to-face surveys included item sets designed to measure 13 *latent* variables of empowerment that conceptually captured claims on critical resources and major domains of agency (Table 2). *Economic resources* was captured with two item sets measuring access to and use of financial resources (8 items) and access to credit (5 items). *Technological resources* was captured with one item set measuring the use of information and communication technologies (ICT, 6 items). *Intrinsic economic, reproductive, bodily, and time-use agency* was captured with four item sets measuring the endorsement of women’s freedom to choose livelihoods (5 items), endorsement of women’s freedom to choose family formation (4 items), rejection of women’s subjection to sexual harassment (5 items), and rejection of gender inequality in time spent on paid and unpaid activities (4 items). Instrumental time-use and economic agency was captured with two item sets measuring the extent of influence over own time

allocation (6 items) and decision-making and control over income (4 items). *Collective agency in organizations* was captured with two item sets measuring confidence to participate in community organizations (5 items) and participation and leadership in community organizations (5 items). *Collective agency in communities* was captured with two item sets measuring perceptions of women’s and men’s community engagement, each with 9 items. Two measures—ownership and tenure security of land and other property and ownership of a mobile phone—were included in the WEMNS module but were not psychometrically assessed, as they were considered observed (non-latent) variables.

#### Validation module.

A validation module also was developed to assess the construct validity of final, maximum a posteriori factor scores [29] for all 13 dimensions of women’s empowerment that were derived from final models (see Analysis, below). The questions included in this validation module were extracted from the World Values Survey and were designed to measure basic needs, resources, agency, and subjective well-being. Basic needs was measured as the self-reported frequency of going without enough food in the last 12 months (often = 1; never = 4). Resources was measured as the self-reported frequency of obtaining information from a mobile phone, and separately, the internet (never = 1; daily = 5). Intrinsic agency was assessed as the self-reported extent of justifying divorce, and separately, not justifying wife beating (never = 1; always = 4). Instrumental agency was assessed as the self-reported extent of: choice and control over one’s life (none = 1; great deal = 4), input into decisions about household income and spending (none = 1; most = 3), and making one’s own decisions about household income and spending (none = 1; high = 4). Subjective well-being was measured as self-rated health (very poor = 1; very good = 5), happiness (not at all = 1; very happy = 4), life satisfaction (completely dissatisfied = 1; completely satisfied = 4), and satisfaction with household financial situation (completely dissatisfied = 1; completed satisfied = 4).

#### Survey platform.

Team members developed a customized data system using World Bank Survey Solutions software for computer-assisted personal interviews (CAPI) (https://mysurvey.solutions). Interviewers administered a household-level module to the primary respondent, preferably the household head. Survey Solutions then was programmed to select randomly one woman and one man 18–64 years from the roster for face-to-face administration of the WEMNS module and a validation module (S1 File).

#### Analysis.

All analyses were conducted using STATA Version 17 (StataCorp, College Station, Texas, USA) and MPlus version 8.6 (Muthén & Muthén, Los Angeles, California, USA). In step 1, we performed univariate analysis to assess the distributions and item-level missingness of demographic variables and all items in the WEMNS module that were included in the psychometric analysis. We also estimated tetrachoric and polychoric correlations between all binary or ordinal WEMNS items, within and between theoretical empowerment constructs.

In step 2, we performed exploratory factor analysis (EFA) in a random split-half sample of the pooled (three-country) dataset (n_1_ = 2,401). We analyzed each WEMNS item set separately (Table 2), resulting in 13 EFAs. Our decision to examine each item set separately during this developmental stage was guided by analytical goals and practical empirical considerations based on initial EFAs with all 75 items (S1 File). Specifically, we aimed for a nuanced individual analysis and validation of all 13 dimensions of women’s empowerment. This approach made the various analyses and interpretations more feasible and ensured that the results were more interpretable, accessible, and useful for researchers, policymakers, and stakeholders. We performed the separate EFAs with means and variance adjusted weighted least squares estimators, which is suitable for binary and ordinal variables [30]. EFA models were fit using geomin (an oblique) rotation to simplify interpretation of the factor structure [27]. Criteria to remove an item from an EFA included low factor loadings (<|0.350|), significant negative factor loadings, and theory (e.g., the remaining items could adequately reflect the construct) [31].

In step 3, for each item set, we performed confirmatory factor analysis (CFA) on the next random split-half sample (n_2_ = 2,402) to test the factor structure identified in the EFA. For all EFA and CFA models, we interpreted overall model fit as ‘good’ based on the following indices and thresholds: upper limit of the confidence interval for the root mean squared error of approximation (RMSEA) equal to.07 or.08, comparative fit index (CFI) close to 0.95 or higher, and Tucker–Lewis index (TLI) close to 0.95 or higher [32].

In step 4, for each empowerment factor identified in the EFA and confirmed in the CFA, we conducted two separate multi-group CFAs (MGCFAs) using the CFA sample (N = 2,402) to test for measurement invariance, one MGCFA for genders and one for country settings. In MGCFA, nested CFA models are estimated, in which equality constraints on specific model parameters are added sequentially to test whether the models with and without those constraints are equivalent across groups [33,34]. In the MGCFA models, one loading per latent variable is fixed to 1 to identify the latent variable and establish its scale for cross-group comparisons. In a baseline model, *configural invariance* is observed when the same factor structure (e.g., the same pattern of factor-item relationships) holds [34], providing evidence that the *factor structure* is invariant and that a similar construct of empowerment is observed across groups [35]. For the empowerment factor with ordinal responses, *metric invariance* is tested by constraining all factor loadings (relating items to the underlying construct) to be equal across groups [34,35]. *Scalar invariance* is tested by constraining all factor loadings *and* item thresholds to be equal across groups [34]. In exact invariance testing, *scalar invariance* is required to validly compare and to interpret differences in scores for a latent empowerment construct across groups [35]. For the dimensions of access to/use of financial services and access to credit with binary indicators, we tested configural versus scalar invariance directly, bypassing metric invariance. For the dimension of use of ICT, initial MGCFA models by country settings failed to converge, possibly due to skewed distributions of the responses, so we dichotomized these items and similarly bypassed metric invariance. This decision was due to the use of the weighted least squares mean and variance adjusted (WLSMV) method, which does not permit testing metric invariance with scale factors or residual variances allowed to vary across groups [29].

The first MGCFA series assessed measurement invariance of each item set across genders, and the second across country settings (Bangladesh, Malawi, Nepal). In each case, we assessed the fit of the configural model using the same criteria, as described for the EFAs and CFAs. To assess fit for the nested metric and scalar models, we considered *changes in model-fit statistics* (chi-square difference test Δχ^2^, ∆CFI, ∆RMSEA) with sequentially added equality constraints on model parameters [33,36,37]. We inferred whether each item set was invariant based on a non-significant chi-square difference at p = 0.05 [34], and guidelines for changes in other model fit statistics with increasing equality constraints (∆CFI ≤ 0.002; ∆RMSEA ≤0.007) [36,37].

In step 5, for item sets in which metric or scalar invariance across groups was not observed, we reviewed results from the MGCFAs, including the modification indices for loadings and thresholds, to assess whether MGCFA with partial invariance testing was neccesary. This review allowed us to identify specific model parameters that were non-invariant across groups and to assess invariance among the remaining parameters [33]. For measures demonstrating at least configural invariance but not scalar invariance, we tested partial scalar invariance only for those measures with few modification indices >10 (the default value in Mplus 8 [28]) for the current scales with a small number of items, under the argument that small-scale noninvariance likely has a negligible impact on the estimation of construct-related parameters [38]. In those cases, we released equality constraints across groups one parameter at a time, starting with the parameter having the largest *modification index*, indicating the expected improvement in the overall model χ^2^ if the equality constraint for the parameter was released [33]. We then tested the partial invariance of models across groups, based on the Δχ^2^, ∆CFI and ∆RMSEA.

In step six, we assessed the construct validity of all 13 WEMNS item sets in the pooled sample. We estimated Spearman pairwise associations between the weighted factor score for each WEMNS item set and variables representing *basic needs*, *resources*, *agency*, and *subjective well-being* from the World Values Survey (see Validation Module, above). Factor scores were calculated using the factor loadings from the final CFA models. Expected bivariate relationships are summarized in the supplemental information (S1 File). Conventions to interpret correlation coefficients were used (<0.30 = weak; ≥ 0.30 and <0.50 = moderate; ≥ 0.50 = large [39]. Finally, we calculated Spearman pairwise correlations between scored item sets to confirm that they were distinct and non-redundant.

## Results

### Step 1. Sample characteristics and distributions of WEMNS items

Across settings, participation rates were 85.5%−99.0% for the sample of women and 85.5%−99.8% for the sample of men (Table 3). The mean age was 31.9–35.6 years for women and 33.5–38.5 years for men. The percentages with no schooling were 4.3%−40.1% among women and 3.9%−23.1% among men.

**Table 3:** WEMNS face-to-face survey participant characteristics, by gender and country setting.

	Bangladesh		Malawi		Nepal	
Characteristics	Women	Men	Women	Men	Women	Men
**Eligible**	816	847	798	792	947	947
**Interviewed**	800	800	791	792	810	810
**Response Rate, %**	98.04	94.45	99.00	99.80	85.50	85.50
**No Schooling, %**	21.1	23.1	4.3	3.9	40.1	17.2
**Age in Years, M(SD)**	34.0(11.0)	38.5(12.1)	31.9(11.0)	33.5(11.8)	35.6(11.4)	37.9(12.2)

Note. In Bangladesh, 861 households were contacted to complete 800 interviews for each gender. In Nepal, enumerators first approached senior household members (heads preferred) to gain consent to survey members, and then identified a man and a woman member to interview. From consenting households, 100% of women and 99.6% of men identified agreed to participate; however, 134 households refused to be surveyed, and response rates account for household-level refusal. In Malawi, enumerators approached 790 households, from which eight women and two men identified could not complete the survey, and replacements within households were surveyed.

Table 4 provides the distributions of each of the 75 WEMNS items included in the psychometric analysis. Missingness was extremely rare (<1% of observations) or rare (<7%) for most items (67 of 75), and responses were treated, conditional on observed variables, to be missing at random in the analysis. A majority of the sample reported that they did not own or have a mobile money account, bank account, bank card, or credit card (58% to 99%), and similar percentages of the sample reported not to have used any of these financial services in the prior 12 months (62% to 98%). A majority of participants reported that they could take out a loan from any of the financial institutions mentioned (66% to 74%), except from an ‘other’ NGO program (43%). Use of ICT varied widely by type, with 97% of the sample reporting never use of a landline and 75% reporting daily use of a mobile phone. A majority of participants “fully disagreed” with most items measuring women’s freedom to choose their own livelihoods (54% to 89% across all items) and freedom to choose family formation (59% to 68% across three of four items). In addition, around half or more participants reported that various forms of sexual harassment against women were “usually acceptable” (48% to 67% across all items). Similarly, a majority of participants “fully agreed” with statements acknowledging the existence of gender inequality (62% to 66% across all items). That said, two-fifths to a majority of the sample reported having “a lot of influence” over their own time allocation (42% to 66% across all items). About half or more participants reported having “some” or “a lot” of influence over financial or personal healthcare decisions (48% to 75% across all items). While almost half to a majority of participants reported being “very confident” to participate in community organizations (45% to 71% across all items), a majority did not participate in community organizations (55% to 91% across all items). In general, higher percentages of the sample “fully agreed” with statements regarding men’s community engagement than with statements regarding women’s community engagement (Table 4).

**Table 4:** Distributions of WEMNS, by dimension of the WEMNS module, pooled sample (N = 4,803).

WEMNS Dimensions	Distribution of Response Options: N (%)					
Access to/ use of financial services	Missing	Yes	No			
Do you own or have any of the following? A mobile money account such as E-Sewa	4 (0.1)	2039 (42.4)	2764 (57.5)			
Do you own or have any of the following? A bank account	6 (0.1)	1438 (29.9)	3363 (70.0)			
Do you own or have any of the following? A bank card, ATM card, or debit card	14 (0.3)	504 (10.5)	4289 (89.2)			
Do you own or have any of the following? A credit card	12 (0.3)	42 (0.9)	4753 (98.8)			
In the past 12 months have you ever used any mobile money account to make a payment, buy something, or send money to someone?	9 (0.2)	1815 (37.8)	2983 (62.1)			
In the past 12 months have you ever deposited or received money into or withdrawn money from any bank account?	10 (0.2)	1239 (25.8)	3558 (74.0)			
In the past 12 months have you ever used any bank card, ATM card, or debit card to make a purchase or pay a bill such as a utility bill?	26 (0.5)	177 (3.7)	4604 (95.8)			
In the past 12 months have you ever used any credit card to make a purchase or pay a bill such as a utility bill?	32 (0.7)	69 (1.4)	4706 (97.9)			
**Access to credit**	**Missing**	**Yes**	**No**			
Could you take a loan from any of the following: bank or formal financial institution	12 (0.3)	3164 (65.8)	1631 (33.9)			
Could you take a loan from any of the following: cooperative	18 (0.4)	3326 (69.2)	1463 (30.4)			
Could you take a loan from any of the following: group based micro-finance	32 (0.7)	3586 (74.6)	1189 (24.7)			
Could you take a loan from any of the following: informal credit/savings groups	20 (0.4)	3255 (67.7)	1532 (31.9)			
Could you take a loan from any of the following: other NGO program	132 (2.8)	2044 (42.5)	2631 (54.7)			
**Use of ICT** ^a^	**Missing**	**Daily**	**Weekly**	**Monthly**	**<Monthly**	**Never**
Please say whether you use a radio daily, weekly, monthly, less than monthly, or never	8 (0.2)	911 (18.9)	549 (11.4)	206 (4.3)	229 (4.8)	2904 (60.4)
Please say whether you use a television daily, weekly, monthly, less than monthly, or never	6 (0.1)	1581 (32.9)	742 (15.4)	242 (5.0)	268 (5.6)	1968 (40.9)
Please say whether you use a landline phone daily, weekly, monthly, less than monthly, or never	10 (0.2)	18 (0.4)	10 (0.2)	8 (0.2)	77 (1.6)	4684 (97.4)
Please say whether you use a mobile phone daily, weekly, monthly, less than monthly, or never	5 (0.1)	3598 (74.8)	652 (13.6)	152 (3.1)	108 (2.2)	292 (6.1)
Please say whether you use a computer, laptop, or computer tablet, such as an ipad or notebook computer daily, weekly, monthly, less than monthly, or never	12 (0.2)	151 (3.1)	99 (2.1)	63 (1.3)	82 (1.7)	4400 (91.5)
Please say whether you use the internet daily, weekly, monthly, less than monthly, or never	14 (0.3)	1229 (25.6)	381 (7.9)	90 (1.9)	116 (2.4)	2977 (61.9)
**Endorsement of women’s freedom to choose livelihoods**	**Missing**	**Fully disagree**	**Partly disagree**	**Partly agree**	**Fully** **agree**	
Every woman should be free to choose whether to complete secondary school	5 (0.1)	4285 (89.1)	404 (8.4)	82 (1.7)	31 (0.6)	
Every woman should be free to choose whether to work for pay	7 (0.1)	3753 (78.1)	779 (16.2)	145 (3.0)	123 (2.6)	
Every woman should be free to choose to prioritize her work for pay over domestic duties	6 (0.1)	2571 (53.5)	1303 (27.1)	453 (9.4)	474 (9.9)	
Every woman should be free to choose what to do with any money that she earns	7 (0.1)	3356 (69.8)	1024 (21.3)	259 (5.4)	161 (3.3)	
Every woman should be free to choose to purchase land, a house, or other valuable goods	7 (0.1)	3134 (65.2)	1149 (23.9)	333 (6.9)	184 (3.8)	
**Endorsement of women’s freedom to choose family formation**	**Missing**	**Fully disagree**	**Partly disagree**	**Partly agree**	**Fully** **agree**	
Every woman should be free to choose when to get married	6 (0.1)	3276 (68.1)	872 (18.1)	374 (7.8)	279 (5.8)	
Every woman should be free to choose to divorce or end her marriage	10 (0.2)	1807 (37.6)	1419 (29.5)	746 (15.5)	825 (17.2)	
Every woman should be free to choose whether and when to have children	8 (0.2)	3037 (63.2)	1169 (24.3)	358 (7.4)	235 (4.9)	
Every woman should be free to choose not to have any more children	9 (0.2)	2848 (59.2)	1158 (24.1)	423 (8.8)	369 (7.7)	
**Rejection of women’s subjection to sexual harassment**	**Missing**	**Never acceptable**	**Sometimes acceptable**	**Usually acceptable**		
A man treats a woman as “lesser” because she is a woman, for example, speaks badly, interrupts, or ignores her	32 (0.7)	1440 (30.0)	334 (6.9)	3001 (62.4)		
A man prevents a woman from doing certain kinds of work, even if she wants to	37 (0.8)	1458 (30.3)	1011 (21.0)	2301 (47.9)		
A man spreads unwanted rumors about a woman’s sex life	34 (0.7)	1474 (30.7)	82 (1.7)	3217 (66.9)		
A man tries to have a romantic or sexual relationship with a woman when she doesn’t want it	31 (0.6)	1433 (29.8)	117 (2.4)	3226 (67.1)		
A man offers work-related benefits to a woman with the expectation of receiving sexual favors	36 (0.7)	1446 (30.1)	93 (1.9)	3232 (67.2)		
**Rejection of gender inequality in time spent on paid/unpaid activities**	**Missing**	**Fully** **agree**	**Partly agree**	**Partly disagree**	**Fully** **disagree**	
Compared to a woman, a man can change his daily schedule more easily	8 (0.2)	3106 (64.6)	1226 (25.5)	291 (6.0)	176 (3.7)	
Because of their responsibilities, women generally sleep less than men do	4 (0.1)	3077 (64.0)	1044 (21.7)	343 (7.1)	339 (7.0)	
Because of their responsibilities, women have less leisure time than men do	5 (0.1)	2988 (62.2)	1100 (22.9)	376 (7.8)	338 (7.0)	
Women’s responsibilities take more time than men’s responsibilities do	6 (0.1)	3153 (65.6)	1058 (22.0)	308 (6.4)	282 (5.9)	
**Extent of influence over own time allocation**	**Missing**	**No influence**	**Some influence**	**A lot of influence**		
Influence over the amount of time spent/not spending time on household duties	11 (0.2)	435 (9.0)	1212 (25.2)	3149 (65.5)		
Influence over the amount of time spent/not spending time on caring for household members	99 (2.1)	484 (10.1)	1098 (22.8)	3126 (65.0)		
Influence over the amount of time spent/not spending time on going to the market	39 (0.8)	708 (14.7)	1311 (27.3)	2749 (57.2)		
Influence over the amount of time spent/not spending time on non-agricultural work activities	219 (4.6)	1275 (26.5)	914 (19.0)	2399 (49.9)		
Influence over the amount of time spent/not spending time on agricultural production for sale	297 (6.2)	1440 (30.0)	1038 (21.6)	2032 (42.3)		
Influence over the amount of time spent/not spending time on agricultural production for household consumption	171 (3.6)	889 (18.5)	1123 (23.4)	2624 (54.6)		
**Decision-making and control over income**	**Missing**	**No influence**	**Some influence**	**A lot of influence**		
How much influence do you have regarding control over any source of income, no influence, some influence, or a lot of influence?	19 (0.4)	453 (9.4)	2030 (42.2)	2305 (47.9)		
Please tell me whether you have no influence, some influence, or a lot of influence in decisions about large household purchases?	8 (0.2)	533 (11.1)	2162 (45.0)	2104 (43.8)		
Please tell me whether you have no influence, some influence, or a lot of influence in decisions about minor household purchases?	7 (0.1)	230 (4.8)	1606 (33.4)	2964 (61.7)		
Please tell me whether you have no influence, some influence, or a lot of influence in decisions about your own healthcare?	11 (0.2)	77 (1.6)	1101 (22.9)	3618 (75.3)		
**Confidence to participate in community organizations**	**Missing**	**Not at all confident**	**Somewhat confident**	**Very confident**		
Confidence to participate in government councils or agencies	8 (0.2)	1306 (27.2)	1309 (27.2)	2184 (45.4)		
Confidence to participate in groups that provide local services	10 (0.2)	655 (13.6)	1361 (28.3)	2781 (57.8)		
Confidence to participate in formal and informal savings or credit groups	10 (0.2)	770 (16.0)	1239 (25.8)	2788 (58.0)		
Confidence to participate in groups related to livelihood activities	10 (0.2)	662 (13.8)	1348 (28.0)	2787 (58.0)		
Confidence to participate in other groups	7 (0.1)	352 (7.3)	1024 (21.3)	3424 (71.2)		
**Participation and leadership in community organizations**	**Missing**	**Did not participate**	**Participated, did not lead**	**Leader**		
Participation and leadership in government councils or agencies	8 (0.2)	4361 (90.7)	292 (6.1)	146 (3.0)		
Participation and leadership in groups that provide local services	4 (0.1)	3958 (82.3)	562 (11.7)	283 (5.9)		
Participation and leadership in formal and informal savings or credit groups	9 (0.2)	3185 (66.3)	1281 (26.6)	332 (6.9)		
Participation and leadership in groups related to livelihood activities	7 (0.1)	4091 (85.1)	480 (10.0)	229 (4.8)		
Participation and leadership in other groups	6 (0.1)	2657 (55.3)	1395 (29.0)	749 (15.6)		
**Perceptions of women’s community engagement**	**Missing**	**Fully disagree**	**Partly disagree**	**Partly agree**	**Fully** **agree**	
Women can really understand what is going on with your community	11 (0.2)	248 (5.2)	338 (7.0)	1652 (34.4)	2558 (53.2)	
Women have a pretty good understanding of the important issues that face your community^b^	1599 (33.3)	226 (4.7)	391 (8.1)	1517 (31.6)	1074 (22.3)	
Women have the ability to participate effectively in community activities	16 (0.3)	171 (3.6)	294 (6.1)	1541 (32.1)	2785 (57.9)	
Women have the ability to participate effectively in decision-making	12 (0.2)	194 (4.0)	345 (7.2)	1674 (34.8)	2582 (53.7)	
Women’s opinion is important because it could someday make a difference in your community^b^	1596 (33.2)	116 (2.4)	188 (3.9)	1248 (26.0)	1659 (34.5)	
There are plenty of ways for women to have a say in what your community does^b^	1602 (33.3)	223 (4.6)	324 (6.7)	1532 (31.9)	1126 (23.4)	
It is important to women that women actively participate in local women’s issues	15 (0.3)	112 (2.3)	192 (4.0)	1393 (29.0)	3095 (64.4)	
Most community leaders would listen to women	17 (0.3)	490 (10.2)	546 (11.4)	1636 (34.0)	2118 (44.1)	
It is important to women to participate in local activities^b^	1594 (33.2)	148 (3.1)	205 (4.3)	1242 (25.8)	1618 (33.7)	
**Perceptions of men’s community engagement**	**Missing**	**Fully disagree**	**Partly disagree**	**Partly agree**	**Fully** **agree**	
Men can really understand what is going on with your community	11 (0.2)	73 (1.5)	130 (2.7)	1020 (21.2)	3573 (74.3)	
Men have a pretty good understanding of the important issues that face your community^b^	1596 (33.2)	22 (0.5)	92 (1.9)	910 (18.9)	2187 (45.5)	
Men have the ability to participate effectively in community activities	15 (0.3)	28 (0.6)	76 (1.6)	962 (20.0)	3726 (77.5)	
Men have the ability to participate effectively in decision-making	14 (0.3)	39 (0.8)	88 (1.8)	1031 (21.4)	3635 (75.6)	
Men’s opinion is important because it could someday make a difference in your community^b^	1600 (33.3)	14 (0.3)	50 (1.0)	830 (17.3)	2313 (48.1)	
There are plenty of ways for men to have a say in what your community does^b^	1600 (33.3)	20 (0.4)	59 (1.2)	964 (20.0)	2164 (45.0)	
It is important to men that men actively participate in local women’s issues	19 (0.4)	29 (0.6)	96 (2.0)	1015 (21.1)	3648 (75.9)	
Most community leaders would listen to men	21 (0.4)	115 (2.4)	207 (4.3)	1356 (28.2)	3108 (64.7)	
It is important to men to participate in local activities^b^	1593 (33.1)	12 (0.2)	52 (1.1)	911 (18.9)	2239 (46.6)	

^a^The response options for the item set on Use of ICT were dichotomized to Ever Used/ Never Used for the MGCFA by country.

^b^These items were not administered in Malawi, and their responses were assumed missing at random in the analysis.

### Steps 2–3. Factor analyses in pooled sample

For a majority of the 13 item sets intended to reflect single dimensions of empowerment, model fit statistics were adequate, with the upper bound of the confidence interval for the RMSEA being ≈0.08 or lower and the CFI and TLI being ≈0.95 or higher. Moreover, the item-specific factor loadings in all cases except two equaled or exceeded 0.35 (Table 5). Item sets for intrinsic economic, reproductive, and time-use agency and for intrinsic collective agency in organizations were the only ones for which one or more model fit statistic in the EFA and/or CFA were not close to the recommended thresholds.

**Table 5:** Results from Exploratory Factor Analyses (EFA) and Confirmatory Factor Analyses (CFA) for each WEMNS item set administered in face-to-face surveys, random split-half samples from all participants across Bangladesh, Malawi, and Nepal.

	EFA (n_1_ = 2401)				CFA (n_2_ = 2402)			
WEMNS Dimensions	RMSEA (90%CI)	CFI	TLI	Loadings Range	RMSEA (90%CI)	CFI	TLI	Loadings Range
**Resources**								
**Access to/ use of financial services**	0.033 (0.025-0.041)	0.959	0.943	0.412-0.937	0.027 (0.019-0.036)	0.964	0.949	0.384-0.990
**Access to credit**	0.012 (0.000-0.033)	0.995	0.990	0.613-0.975	0.012 (0.000-0.032)	0.998	0.996	0.654-0.939
**Use of ICT**	0.024 (0.011-0.037)	0.946	0.894	0.418-0.835	0.032 (0.020-0.045)	0.934	0.890	0.312-0.874
**Intrinsic Agency**								
**Endorsement of women’s freedom to choose livelihoods**	0.038 (0.023-0.055)	0.914	0.827	0.434-0.794	0.032 (0.016-0.049)	0.938	0.876	0.538-0.857
**Endorsement of women’s freedom to choose family formation**	0.299 (0.276-0.323)	0.000	0.000	0.600-0.791	0.056 (0.033-0.081)	0.942	0.827	0.573-0.819
**Rejection of women’s subjection to sexual harassment**	0.000 (0.000-0.020)	1.000	1.001	0.923-1.000	0.000 (0.000-0.021)	1.000	1.000	0.920-1.007
**Rejection of gender inequality in time spent on paid, unpaid activities**	0.077 (0.054-0.102)	0.832	0.495	0.361-0.749	0.049 (0.027-0.075)	0.865	0.596	0.430-0.742
**Instrumental Agency**								
**Extent of influence over own time allocation**	0.037 (0.025-0.049)	0.961	0.936	0.699-0.878	0.039 (0.027-0.051)	0.966	0.944	0.751-0.857
**Decision-making and control over income**	0.035 (0.013-0.063)	0.997	0.991	0.688-0.921	0.066 (0.043-0.091)	0.990	0.969	0.678-0.885
**Collective Agency**								
**Confidence to participate in community organizations**	0.029 (0.013-0.046)	0.915	0.830	0.689-0.898	0.041 (0.025-0.057)	0.819	0.637	0.675-0.875
**Participation and leadership in community organizations**	0.000 (0.000-0.021)	1.000	1.000	0.416-0.715	0.000 (0.000-0.021)	1.000	1.000	0.334-0.762
**Perceptions of women’s community engagement**	0.022 (0.014-0.029)	0.983	0.977	0.556-0.734	0.036 (0.029-0.043)	0.958	0.944	0.481-0.755
**Perceptions of men’s community engagement**	0.029 (0.022-0.036)	0.992	0.989	0.536-0.816	0.026 (0.019-0.034)	0.971	0.962	0.558-0.802

Notes. RMSEA = Room Mean Square Error of Approximation; CFI = Comparative Fit Index; TLI = Tucker-Lewis Index.

### Step 4. Measurement invariance testing across gender and country setting

In MGCFA of the same 13 item sets, configural invariance (Yes or Close to) across gender was observed for nine item sets, such that all instrumental, and collective agency constructs appear to have been understood similarly across gender in this sample (Table 6). For all three measures of resources—access to and use of financial services, access to credit, and use of ICT—configural invariance was not observed. Similarly, for two measures of intrinsic agency—endorsement of women’s freedom to choose livelihoods and endorsement of women’s freedom to choose family formation—configural invariance was not observed. Metric invariance was observed for seven measures—two for intrinsic agency, one for instrumental agency, and four for collective agency. Scalar invariance was observed for only one measure, rejection of women’s subjection to sexual harassment (Table 6).

**Table 6:** Results of measurement invariance testing across gender for each WEMNS item set administered in the face-to-face survey, CFA sample of participants from Bangladesh, Malawi, and Nepal (n_2_ = 2402).

Model	χ2	RMSEA	CFI	TLI	Δχ2 (diff test)	ΔRMSEA	ΔCFI	Data support invariance
**Resources**								
**Access to/ Use of financial services** ^a^								
Configural	3213.176	0.182	0.797	0.716	--	--	--	**No**
Configural v scalar	2947.728	0.162	0.814	0.774	<0.001	−0.020	0.017	**N/A**
**Access to credit** ^a^								
Configural	191.245	0.087	0.982	0.964	--			**No**
Configural v scalar	222.684	0.082	0.979	0.968	<0.001	−0.005	−0.003	**N/A**
**Use of ICT**								
Configural	241.628	0.096	0.865	0.798	--			**No**
Configural v metric	242.794	0.085	0.868	0.841	<0.001	0.011	0.003	**N/A**
Metric v scalar	269.630	0.065	0.864	0.909	0.0064	0.020	−0.004	**N/A**
**Intrinsic Agency**								
**Endorsement of women’s freedom to choose livelihoods**								
Configural	132.272	0.101	0.960	0.920	--	--	--	**No**
Configural v metric	150.49	0.090	0.955	0.936	<0.001	−0.011	−0.005	**N/A**
Metric v scalar	217.73	0.075	0.938	0.956	<0.001	−0.015	−0.017	**N/A**
**Endorsement of women’s freedom to choose family formation**								
Configural	55.472	0.104	0.985	0.956	--	--	--	**No**
Configural v metric	53.099	0.074	0.987	0.977	0.0663	−0.03	0.002	**N/A**
Metric v scalar	65.801	0.047	0.986	0.991	0.1404	−0.027	−0.001	**N/A**
**Rejection of women’s subjection to sexual harassment**								
Configural	31.550	0.042	1.000	1.000	--	--	--	**Yes**
Configural v metric	39.675	0.039	1.000	1.000	0.0138	−0.003	0.000	**Yes**
Metric v scalar	59.195	0.036	1.000	1.000	0.0139	−0.003	0.000	**Yes**
**Rejection of gender inequality in time allocated to paid, unpaid activities**								
Configural	41.773	0.089	0.984	0.953	--	--	--	**Yes**
Configural v metric	46.192	0.068	0.984	0.972	0.0093	−0.021	0.000	**Yes**
Metric v scalar	197.255	0.091	0.926	0.951	<0.001	0.023	−0.058	**No**
**Instrumental Agency**								
**Extent of influence over own time allocation**								
Configural	107.818	0.065	0.986	0.976	--	--	--	**Yes**
Configural v metric	128.128	0.062	0.983	0.978	<0.001	−0.003	−0.003	**No**
Metric v scalar	176.749	0.059	0.977	0.980	<0.001	−0.003	−0.006	**N/A**
**Decision-making and control over income**								
Configural	52.078	0.100	0.990	0.970	--	--	--	**Yes**
Configural v metric	39.030	0.062	0.993	0.989	0.7000	−0.038	0.003	**Yes**
Metric v scalar	201.864	0.106	0.961	0.966	0.0000	0.044	−0.032	**No**
**Collective Agency**								
**Confidence to participate in community organizations**								
Configural	178.014	0.118	0.982	0.963	--	--	--	**Close to**
Configural v metric	156.935	0.092	0.984	0.978	<0.001	−0.026	0.002	**Yes**
Metric v scalar	198.018	0.080	0.981	0.983	<0.001	−0.012	−0.003	**No**
**Participation and leadership in community organizations**								
Configural	6.220	0.000	1.000	1.000	--	--	--	**Yes**
Configural v metric	8.972	0.000	1.000	1.000	0.6315	0.000	0.000	**Yes**
Metric v scalar	239.144	0.088	0.820	0.844	<0.001	0.088	−0.180	**No**
**Perceptions of women’s community engagement**								
Configural	83.766	0.078	0.989	0.978	--	--	--	**Yes**
Configural v metric	75.548	0.061	0.991	0.987	0.163	−0.017	0.002	**Yes**
Metric v scalar	129.215	0.055	0.985	0.989	<0.001	−0.006	−0.006	**No**
**Perceptions of men’s community engagement**								
Configural	54.270	0.061	0.990	0.980	--	--	--	**Yes**
Configural v metric	47.492	0.045	0.992	0.989	0.3118	−0.016	0.002	**Yes**
Metric v scalar	84.319	0.041	0.987	0.991	<0.001	−0.004	−0.005	**No**

^a^Metric invariance was not assessed because the response options were binary rather than ordinal and the WLSMV estimation method was used.

Note: N/A indicates that invariance was not assessed due to prior non-invariance (e.g., poor configural or metric fit).

In MGCFA to assess measurement invariance across country settings, configural invariance (Yes or Close to) was observed for 9 of the 10 item sets for which MGCFA models converged (Table 7), suggesting that most WEMNS constructs for resources and agency appear to be understood in similar ways across country settings in this sample. As noted above, response options for the item set on use of ICT were dichotomized for the MGCFA across country settings, and metric invariance was not assessed. Metric invariance was observed for four item sets—endorsement of women’s freedom to choose livelihoods, rejection of gender inequality in time allocated to paid/unpaid activities, extent of influence over own time allocation, and decision-making control over income. Scalar invariance was not observed for any item set.

**Table 7:** Results of measurement invariance testing across country setting for each WEMNS item set administered in face-to-face surveys, CFA sample of participants from Bangladesh, Malawi, and Nepal (n_2_ = 2402).

Model	χ2	RMSEA	CFI	TLI	Δχ2 (diff test)	ΔRMSEA	ΔCFI	Data support invariance
**Resources**								
**Access to/ Use of financial services** ^a^								
Configural^a^	454.932	0.091	0.964	0.950	--	--	--	**Close to**
Configural v scalar	1208.582	0.140	0.898	0.880	<0.001	0.049	−0.066	**No**
**Access to credit** ^a^								
Configural	69.697	0.068	0.992	0.984	--	--	--	**Yes**
Configural v scalar^b^	--	--	--	--	--	--	--	--
**Use of ICT** ^a^								
Configural	80.187	0.050	0.969	0.948	--	--	--	**Yes**
Configural v scalar	599.363	0.142	0.669	0.575	<0.001	0.092	−0.300	**No**
**Intrinsic Agency**								
**Endorsement of women’s freedom to choose livelihoods**								
Configural	118.333	0.093	0.979	0.957	--	--	--	**Close to**
Configural v metric	132.32	0.077	0.977	0.970	<0.001	−0.016	−0.002	**Yes**
Metric v scalar	1318.325	0.176	0.737	0.845	<0.001	0.099	−0.240	**No**
**Endorsement of women’s freedom to choose family formation**								
Configural	67.131	0.113	0.993	0.978	--	--	--	**No**
Configural v metric	72.499	0.079	0.993	0.989	0.0049	−0.034	0.000	**N/A**
Metric v scalar	939.596	0.183	0.893	0.943	<0.001	0.104	−0.100	**N/A**
**Rejection of women’s subjection to sexual harassment** ^b^								
**Rejection of gender inequality in time allocated to paid, unpaid activities**								
Configural	34.759	0.077	0.989	0.967	--	--	--	**Yes**
Configural v metric	43.550	0.057	0.988	0.982	0.0285	−0.020	−0.001	**Yes**
Metric v scalar	874.543	0.176	0.680	0.831	<0.001	0.119	−0.308	**No**
**Instrumental Agency**								
**Extent of influence over own time allocation**								
Configural	125.151	0.068	0.980	0.966	--	--	--	**Yes**
Configural v metric	140.634	0.059	0.979	0.974	0.0021	−0.009	−0.001	**Yes**
Metric v scalar	293.488	0.071	0.952	0.963	<0.001	0.012	−0.027	**No**
**Decision-making and control over income**								
Configural	46.763	0.092	0.992	0.977	--	--	--	**Close to**
Configural v metric	61.647	0.072	0.991	0.986	0.0010	−0.020	−0.001	**Yes**
Metric v scalar	594.39	0.176	0.893	0.916	<0.001	0.104	0.098	**No**
**Collective Agency**								
**Confidence to participate in community organizations**								
Configural	96.300	0.082	0.991	0.982	--	--	--	**Yes**
Configural v metric	169.948	0.089	0.984	0.979	<0.001	0.007	−0.007	**No**
Metric v scalar	1183.5	0.187	0.876	0.910	<0.001	0.098	0.108	**N/A**
**Participation and leadership in community organizations** ^b^								
**Perceptions of women’s community engagement**								
Configural	84.077	0.076	0.988	0.976	--	--	--	**Yes**
Configural v metric	283.233	0.119	0.955	0.941	<0.001	0.043	−0.033	**No**
Metric v scalar	1041.135	0.156	0.827	0.899	<0.001	0.037	0.128	**N/A**
**Perceptions of men’s community engagement** ^b^								

^a^Metric invariance was not assessed because the response options were binary rather than ordinal and the WSLVM estimation method was used.

^b^The model failed to converge.

Note: N/A indicates that invariance was not assessed due to prior non-invariance (e.g., poor configural or metric fit).

### Step 5. Partial invariance testing across gender and country setting

To consider testing for partial scalar invariance across gender, seven measures were eligible, as the results of MGCFA across gender indicated at least configural but not scalar invariance (Table 6). For six of these measures, the presence of many large modification indices suggested many potential sources of mis-fit; therefore, we determined that performing partial invariance testing was not appropriate for these measures. We assessed partial scalar invariance for one measure, decision-making and control over income; however, the data did not support partial invariance (results available upon request). Given that the data did not support scalar invariance of any of the WEMNS item sets across country settings, and in view of the large changes in CFI and RMSEA indicating pervasive structural differences (Table 7), we determined that it was not appropriate to pursue partial invariance testing across country settings.

Table 8 summarizes the results of invariance testing of WEMNS item sets.

**Table 8:** Results of partial invariance testing of WEMNS item sets.

Item Set	Level of Invariance Achieved		Data support scalar invariance	
	Gender	Country	Gender	Country
**Resources**				
Access to use of financial services	None	**Configural**	No	No
Access to credit	None	**Configural**	No	No
Access to/ use of resources through ICT	None	**Configural**	No	No
**Intrinsic Agency**				
Endorsing women’s freedom to choose livelihoods	None	**Metric**	No	No
Endorsing women’s freedom to choose family formation	None	None	No	No
Rejection of women’s subjection to sexual harassment	**Scalar**	None	**Yes**	No
Awareness of gender inequality in time allocated to paid/unpaid activities	**Metric**	**Metric**	No	No
**Instrumental Agency**				
Ability to influence own time allocation	**Configural**	**Metric**	No	No
Decision-making and control over income	**Metric**	**Metric**	No	No
**Collective Agency Measures**				
Confidence to participate in community organizations	**Metric**	**Configural**	No	No
Instrumental organizational collective agency	**Metric**	None	No	No
Perceptions of women’s community engagement	**Metric**	**Configural**	No	No
Perceptions of men’s community engagement	**Metric**	None	No	No

### Step 6. Construct validity of derived factor scores for WEMNS item sets

Overall, Spearman correlations among the weighted factor scores for each item set were weak (Table 9): 58 (87.9%) of 66 correlation coefficients were <0.30, seven (10.6%) were ≥0.30 and <0.50, and one (1.5%) was ≥ 0.50, with the maximum correlation being 0.52 between endorsement of women’s freedom to choose her preferences in livelihoods and to choose her preferences regarding family formation. In other words, all item sets were distinct and most were weakly correlated, further justifying the decision to analyze them separately.

**Table 9:** Spearman pairwise correlations between WEMNS factor scores derived from CFA models.

	WEMNS Dimensions (factor scores derived from final CFA model)											
WEMNS Dimensions (factor scores derived from final CFA model)	Use of financial services	Access to credit	Decision-making and control over income	Access to/ use of ICT	Endorsement of women’s freedom to choose livelihoods	Endorsement of women’s freedom to choose family formation	Awareness of gender inequalities in time allocated to paid, unpaid activities	Ability to influence own time allocation	Confidence to participate in community organizations	Participation, leadership in community organizations	Perceptions of women’s community engagement	Perceptions of men’s community engagement
**Use of financial services**	1											
**Access to credit**	0.1432	1										
**Decision-making and control over income**	0.1832	0.2174	1									
**Access to/ use of ICT**	0.0896	0.0232	−0.0041	1								
**Endorsement of women’s freedom to choose her preferences in livelihoods**	−0.0335	0.0195	−0.0156	−0.0611	1							
**Endorsement of women’s freedom to choose her preferences in family formation**	−0.0167	0.0171	−0.0466	−0.0493	0.5225	1						
**Awareness of gender inequalities in time allocated to paid, unpaid activities**	−0.0419	0.0932	0.0426	−0.0062	0.2340	0.1795	1					
**Ability to influence own time allocation**	0.1216	0.2222	0.3636	0.0487	−0.0033	−0.0315	0.0958	1				
**Confidence to participate in community organizations**	0.1477	0.1479	0.1967	−0.0046	0.2746	0.2346	0.2155	0.1978	1			
**Participation, leadership in community organizations**	0.2027	0.0624	0.1421	0.0167	0.1092	0.1294	−0.0131	0.1752	0.3570	1		
**Perceptions of women’s community engagement**	0.0297	−0.0369	0.0542	−0.0502	0.4487	0.3804	0.2287	−0.0056	0.4506	0.2441	1	
**Perceptions of men’s community engagement**	0.0401	0.0578	0.1421	0.0372	0.2149	0.2013	0.2424	0.1587	0.3272	0.1490	0.4156	1

Note: Factor scores could not be computed for the item set representing rejection of women’s subjection to sexual harassment.

Spearman correlations between the weighted factor score for each WEMNS item set and variables for basic needs, resources, agency, and subjective well-being also were weak (Table 10): 139 (96.5%) of 144 correlation coefficients were <0.30, four (2.8%) were ≥0.30 and <0.50, and one (0.01%) was ≥ 0.50. The five moderate-to-high correlations conceptually aligned. Two were between the score for decision-making and control over income and variables for input and decision-making over income. Another two were between the score for access to/ use of ICT and variables for the frequency of obtaining information from mobile phones or the internet. The fifth was between the score for use of financial services (including mobile money) and the variable for the frequency of obtaining information from the internet.

**Table 10:** Spearman’s pairwise correlations of WEMNS factor scores derived from CFA models and constructs from the world values survey.

	Self-report measures to assess convergent or discriminant construct validity											
	Basic Needs	Resources		Agency					Subjective Well-Being			
WEMNS Dimensions (factor scores derived from final CFA model)	Freq went w/o food last 12 months	Freq of obtaining info from mobile phone	Freq of obtaining info from internet	Extent divorce justified	Extent wife beating not justified	Extent of choice over one’s life	Input into decisions about HH income	Ability to make own decisions about HH income	Happiness	Health	Life satisfaction	Satisfaction with HH financial situation
**Use of financial services**	0.1067	0.2991	0.3372	0.0414	0.1093	0.1051	0.1796	0.1714	−0.0006	0.0195	0.0223	0.0856
**Access to credit**	0.1254	−0.0314	0.0484	−0.1059	0.1491	0.0832	0.1948	0.2742	0.0550	0.0296	0.1482	0.1447
**Decision-making and control over income**	−0.0148	0.0189	0.0005	−0.0351	0.0506	0.2522	0.5071	0.4615	0.0640	0.0254	0.1072	0.1191
**Access to/ use of ICT**	0.0568	0.0463	0.1661	−0.0525	0.1004	−0.0058	0.0586	0.0750	0.0588	0.0173	0.0735	0.0899
**Endorsement of women’s freedom to choose her preferences in livelihoods**	−0.1511	−0.0132	−0.0845	0.1856	−0.1091	0.1104	−0.0179	−0.0318	0.0323	−0.0611	−0.0034	−0.0074
**Endorsement of women’s freedom to choose her preferences in family formation**	−0.1521	0.0257	−0.0639	0.2365	−0.1530	0.0610	−0.0418	−0.1062	0.0066	−0.0571	−0.0202	0.0023
**Awareness of gender inequalities in time allocated to paid, unpaid activities**	−0.1386	−0.0707	−0.1223	0.0035	−0.0375	0.0757	0.0723	0.0713	0.0384	−0.0098	0.0433	0.0370
**Ability to influence own time allocation**	0.0208	−0.0632	−0.0316	−0.0835	0.0799	0.1697	0.3183	0.3505	0.0023	−0.0087	0.1090	0.1283
**Confidence to participate in community organizations**	−0.2882	0.1141	−0.0200	0.2632	−0.2026	0.2482	0.1739	0.0801	0.0268	−0.0863	−0.0446	−0.0666
**Participation, leadership in community organizations**	−0.1168	0.1222	0.0756	0.1293	−0.0442	0.1348	0.1065	0.0858	0.0152	−0.0646	0.0005	−0.0103
**Perceptions of women’s community engagement – Bangladesh and Nepal only**	−0.2828	0.0728	−0.0518	0.2369	−0.2005	0.2022	0.0699	−0.0331	0.0017	−0.0948	−0.0812	−0.0821
**Perceptions of men’s community engagement – Bangladesh and Nepal only**	−0.1489	−0.0286	−0.0640	0.0658	−0.0748	0.1469	0.1469	0.0786	0.0330	−0.0102	0.0390	−0.0032

Note: Factor scores could not be computed for the item set representing rejection of women’s subjection to sexual harassment.

## Discussion

This paper presents findings from the psychometric assessment of the WEMNS module, a concise yet comprehensive survey module measuring domains of women’s empowerment that align with theoretical constructs of resources and agency as well as key gender-related SDG targets and indicators. No such module exists for use *across* NSOs and global survey platforms to compare national changes in a common measure of women’s empowerment. Such a module would fill a major global gap in national-level data on women’s empowerment [22] and would enable researchers to harmonize analyses of the relationships of women’s empowerment with other SDGs collected by different survey platforms (Table 1). Such data would provide the evidence needed for governments and donors to better target investments to advance women’s empowerment for the benefit of women, families, and societies.

Results presented here suggest cautious optimism regarding the WEMNS module’s capacity to fill critical data gaps. In the pooled sample, most WEMNS item sets had clear factor structures that aligned with theoretical dimensions of empowerment and SDG targets and indicators. Configural invariance (e.g., items in a set load on a single latent variable in the same pattern across groups) was observed for nine of 13 item sets across gender and nine of 13 item sets across country setting. Thus, most WEMNS item sets capture common constructs of empowerment across gender and diverse country settings. However, scalar invariance was observed for only one item set across gender and no item sets across country setting, suggesting that the item sets did not measure the same empowerment constructs in equivalent ways across gender or across country setting. Most WEMNS constructs would benefit from further refinement and testing to be suitable for face-to-face data collection and cross-cultural comparative analysis.

Notably, we assessed the psychometric properties of item sets in the face-to-face WEMNS module separately. This decision was guided by the many item sets, evidence from the phone-survey analyses [26], initial EFA findings in which multidimensional models had poor fit or failed to converge (S1 File), and findings here that the factors represented by single item sets were weakly correlated (Table 9). These practical and empirical considerations favored assessing psychometrically WEMNS item sets separately and deriving separate scores for each item set. None of the psychometric analyses, therefore, provided empirical support for creating a combined scale, based on the WEMNS empowerment factor scores. Moreover, the separate analyses of the item sets here do not provide guidance about the empirical performance of the counting-based WEMNS index, which combines resource and agency indicators, treating them as directly observed (non-latent) variables [24].

Notably, the samples for our analyses were not drawn in identical ways (S1 File). For developing and testing scales, however, representative samples are not initially required, provided that the study samples are large and diverse enough for cross-group comparisons. Also, the long-term goal is to recommend a scale for cross-gender, cross-cultural, and cross-time comparison by testing and confirming its equivalence across many diverse samples. Still, any point estimates derived from these study samples may not be representative of the geographic areas from where they were drawn.

These considerations notwithstanding, development of the WEMNS module was informed by a multi-disciplinary, multi-institutional, multi-cultural team. The WEMNS module benefitted from a review of questionnaires across multiple surveys platforms, inclusion of previously validated questions [40,41] , knowledge from experts in the field, local knowledge from multiple rounds of testing and piloting, and a large face-to-face survey in urban/rural samples in three diverse settings. The team also followed best practices for the development and psychometric assessment of new scales [42]. This level of rigor is rare in the development of measures for women’s empowerment. Finally, the WEMNS module covers major domains of resources and agency that align with theories of empowerment and three gender-related SDGs, covering 13 distinct empowerment-related constructs with only 75 survey items here and 64 items at most for the counting-based index, detailed elsewhere [24]. Its concise, yet comprehensive structure and cross-sectoral applicability among adult women and men make it unique among measures of women’s empowerment.

The results of this analysis suggest the benefit of further refinement of item sets in the WEMNS module, particularly if their intended use is the comparison of estimates across men and women or across country settings. As a next step, the WEMNS module has been integrated into household surveys as part of the 50x2030 Initiative, with suggested refinements from country partners. We encourage further investments in repeated, nationally representative surveys to ensure that measures of women’s empowerment can be used validly to compare derived estimates across countries and over calendar time to monitor SDG5 and gender-related SDGs globally. We also recommend investments to include a refined WEMNS module in cross-cultural panel studies of adult women and men to understand the comparability of measures of empowerment over the life course, trajectories of empowerment over age, and reciprocal influences with indicators of demographics; household needs, socioeconomic status, and welfare; individual beliefs and community social norms; as well as women’s, children’s, and family health and well-being.

## Conclusion

The WEMNS module offers a concise, comprehensive measure of women’s and men’s empowerment that shows potential for use to monitor SDG5 and other gender-related SDGs. Further refinement of item sets is advised, especially if the item sets are to be used for comparative purposes. The field of women’s empowerment would benefit from similarly rigorous studies to develop and to assess measures for women’s empowerment to ensure that cross-cultural, cross-gender, cross-group, cross-program, and cross-time comparisons are valid and meaningfully inform equitable national policies and investments.

## Supporting information

S1 File Setting-specific Sample Designs; WEMNS face-to-face survey module; Initial exploratory factor analysis of WEMNS; Expected relationships of WEMNS derived factor scores with external measures [43–45]. (DOCX)
