# Supplementary material for: Women’s Empowerment Metric for National Statistical Systems (WEMNS): Development and psychometric assessment of a face-to-face survey module
Source: PLoS One. 2026 May 8;21(5):e0345742. doi: 10.1371/journal.pone.0345742 (PMC13155635; doi:10.1371/journal.pone.0345742)
Supplement: S1 File — (DOCX) [file pone.0345742.s001.docx]

# **S1. Supporting Information File**

# **Setting-specific sample designs**

In Bangladesh, one division from each region of the country was selected: Rangpur in the north, Khulna in the south, and Mymensingh in the central region. Within each selected division, the latest publicly available census reports were used to develop sampling frames of rural villages and urban informal settlements (43, 44). The sampling plan, then, followed a two-stage stratified cluster random sampling design (45). The study area was divided into six strata (north-rural, north-urban, central-rural, central-urban, south-rural, and south-urban). Twenty primary sampling units (PSUs, villages) were selected from the rural sampling frame, and 20 PSUs (informal settlements with more than 60 households) were selected from the urban sampling frame. A complete household listing was conducted in each selected PSU, and households that confirmed the presence of at least one man and one woman in the target age range were eligible. Twenty eligible households were selected randomly from each PSU to achieve the desired sample size of 800 households. Within each household, one woman and one man between the ages of 18 and 64 were randomly selected using the CAPI program; the final sample comprised 800 households, with 800 men and 800 women interviewed.

In Malawi, the study covered 12 districts across the central and southern regions of the country. The districts were stratified into rural and urban areas. In each district, PSUs were selected randomly proportional to population size. A full household listing was conducted in each selected PSU, identifying eligible households. From the universe of eligible households, 11 core and 11 replacement households (assuming 50% non-response at the household level) were randomly selected from each PSU using STATA statistical software. The actual need for replacement households was about 33%. In total, 792 households and 1583 individual participants were interviewed, of whom 792 were male and 791 were female.

In Nepal, the study sites were Rupandehi and Kapilvastu districts in Lumbini Province of Western Nepal. PSUs were created by splitting or combining formerly delineated wards. A total of 54 PSUs, each with approximately 200 households, were selected. A quick count of households was conducted in selected PSUs to generate a frame of eligible households (at least one woman and one man in the target age range). A unique random seed was set to rank eligible households across the PSUs. Fifteen core and six replacement households were drawn from each PSU, such that 810 core and 324 replacement households were ex-ante randomly selected using Stata Version 17.0. A total of 813 households were interviewed, for a household-level response rate of 85.9%. In total, 1620 participants were interviewed (810 women and 810 men).

# **WEMNS face-to-face survey module**

**September, 2022**

**Table of Contents**

[**Informed Consent of Respondent** X](#_Toc114475213)

[**Section A. Respondent information** X](#_Toc114475212)

[Household questions removed]

[**Section D: Paid and unpaid activities** X](#_Toc114475216)

[**Section E: Participation and leadership in community organizations** XX](#_Toc114475217)

[**Section F: Life transitions and awareness of rights** XX](#_Toc114475218)

[**Section G: Access to services and ownership of resources** XX](#_Toc114475219)

[**Section H: Property ownership** XX](#_Toc114475220)

[**Section I: Decision-making/Control over income** XX](#_Toc114475221)

[**Section J: Access and use of ICT** XX](#_Toc114475222)

[**Section K: Validation** XX](#_Toc114475223)

[**Section J: Sexual Harassment** XX](#_Toc114475224)

[**Section Z: Result of the Interview** XX](#_Toc114475225)

**Informed Consent of Respondent**

Greetings! My name is _________________________ and I work for <SURVEY FIRM>. Together with the International Food Policy Research Institute (IFPRI) we are conducting a study in this area to ask about the experiences and opinions of women and men in your community. Your household has been selected randomly, along with some other households in your community, to participate in this study.

Your participation will consist of an interview with the head of the household or someone who can speak on their behalf, as well as an individual interview with one randomly selected woman and one randomly selected man in the household, who are between ages 18 to 64 years old. The household interview will last about 10 minutes, and each individual interview will last about 30 minutes. The interviews will mainly consist of questions to understand your opinions on important topics. We will also ask you some additional questions about the people who live in your household, characteristics of your house, and the type of assets you own.

We ask for your support by responding to the questions as honestly and fully as possible. This is not an assessment of your efforts and there are no right or wrong answers. Your responses will be kept COMPLETELY CONFIDENTIAL. This means that no one will be able to find out what your responses to these questions were. The answers you provide will not be shared with regulators or any accreditation team. We will use the responses to say things such as, in general people in this area tend to have a certain opinion.

Your participation in this survey is voluntary. If you choose to participate, you may refuse to answer certain questions, or you may stop participating at any time. There is a potential risk of transmitting an illness while conducting face-to-face interviews. To ensure that the risk is minimal, I will be wearing a mask during the interview. I also have a mask available for you if you would like one to wear. We will also sit approximately 1.5 meters away from each other in the open air. There is no other foreseen risk to your participation in this survey.

If you have questions about this survey or study, you may contact any of the following: <local contact information>.

You can ask questions concerning the study, both before agreeing to be involved and during the interview.

Do you consent to participate in this interview? (Interviewer to mark oral response)

□ Yes, I am willing to participate voluntarily

□ No, I am not willing to participate in the survey

**Section A. Respondent information**

| **Sample Identification** | **Name** | | |
| --- | --- | --- | --- |
| A01. Household Number | *Please enter HHID (twice)* | | |
| A02. Region | *Prefilled* | | |
| A03. District | *Prefilled* | | |
| A04. Enumeration Area | *Prefilled* | | |
| A05. Enumerator ID and name |  | | |
| A06 Location: Full address of household |  | | |
| A07. GPS Coordinates | \| Latitude N \|  \|  \| ˚ \|  \|  \| **’** \|  \|  \| **”** \| \| --- \| --- \| --- \| --- \| --- \| --- \| --- \| --- \| --- \| --- \| \| Longitude E \|  \|  \| ˚ \|  \|  \| **’** \|  \|  \| **”** \| | | |
| A08. Respondent name |  | | |
| A09. What type of household is this? (enter code) |  | | |
| A10. Does the household have both a male and female aged 18 to 64? (Yes/No) |  | | |
| A11. Outcome of visit (enter code) | 1^st^ visit: | 2^nd^ visit: | 3^rd^ visit: |
| START_TIME. Start of survey date and time stamp | \| DD \| MM \| YYYY \| HH: \| MM: \| SS \| \| --- \| --- \| --- \| --- \| --- \| --- \|   *Automatically recorded* | | |

| **LANGUAGE**  **(ADAPT TO COUNTRY CONTEXT)**  1 = English  2 = ChiChewa  3 = ChiYao  4 = ChiTimbuka  5.=Bangla  96 = Other (specify) | **TYPE OF HOUSEHOLD**  1 = Male and female adult  2 = Female adult only (END INTERVIEW)  3 = Male adult only (END INTERVIEW)  4 = No male or female adults (END INTERVIEW) | **OUTCOME OF VISIT**  01 = Respondent Available (CONTINUE)  02 = Entire household absent outside the area for extended period of time (END INTERVIEW)  03 = Refused (in general) (END INTERVIEW)  04 = Refused to consent (END INTERVIEW)  05 = Postponed (RETURN FOR CALL-BACK)  06 = No household member at home or no competent respondent at home at time of visit (RETURN FOR CALL-BACK)  07 = Partial (RETURN FOR CALL-BACK)  96 = Other (specify) __________________________ |  |
| --- | --- | --- | --- |

**THE FOLLOWING SECTIONS NEED TO BE ASKED TO THE WOMAN AND MAN RESPONDENTS SEPARATELY**

**Section D: Paid and unpaid activities**

| D_START. Start of Section D date and time stamp: | DD | MM | YYYY | HH: | MM: | SS |
| --- | --- | --- | --- | --- | --- | --- |

| **D00. INTERVIEWER, READ ALOUD:** Now, the interview with the individual respondents will begin. We will speak separately to the selected male and female households members. |
| --- |
| **D00a. If the selected individual is different from the household roster respondent, please re-read the introduction and consent statement.**  **Does the individual respondent consent to participate in this survey?**  Yes =1  No = 2 |

**INTERVIEWER NOTE:** This questionnaire Section is split into sections, each of which has its own instructions. For each set of instructions that indicates "INTERVIEWER, READ ALOUD", read the instructions out loud to the respondent exactly as they are written; do not summarize the instructions. After reading the instructions to the respondent, read each prompt exactly as it is written followed by all response options except "Refuse, Not applicable, and Cannot answer". When you read through the response options, where a response card (showcard) is indicated, show the response card and point to where each option falls on the card to provide the respondent with both a verbal and visual orientation to the options. If the respondent does not understand the statement the first time you read it, you can read the statement exactly as it is written up to two more times. Do not give any of your own examples or provide any of your own clarifications about the statements. Based on the respondent's answer, select the response choice that best aligns with the stated options.

| **(USE SHOWCARD #1 - Optional)** | |
| --- | --- |
| **D01. INTERVIEWER, READ ALOUD:** Now, I am going to read several statements about **PERCEPTIONS OF DIFFERENCES WITHIN A HOUSEHOLD**. Please listen to each statement and **indicate [show response card and point to the related response option on the card] - whether you fully disagree, partly disagree, partly agree, or fully agree with the statement**. We will use response cards for this survey because sometimes it is difficult to remember all the response options, and some people find it easier when we use a card as a reminder. There are no right or wrong answers, only your opinion matters. Let’s begin. | |
| D01A. Compared to a woman, a man can **change his daily schedule more easily**. | 0 = Fully disagree  1 = Partly disagree  2 = Partly agree  3 = Fully agree  97 = DO NOT READ: Don’t know  98 = DO NOT READ: Refused  99 = DO NOT READ: Not applicable |
| D01B. Because of their responsibilities, women **generally sleep less** than men do. | 0 = Fully disagree  1 = Partly disagree  2 = Partly agree  3 = Fully agree  97 = DO NOT READ: Don’t know  98 = DO NOT READ: Refused  99 = DO NOT READ: Not applicable |
| D01C. Because of their responsibilities, women **have less leisure time** than men do. | 0 = Fully disagree  1 = Partly disagree  2 = Partly agree  3 = Fully agree  97 = DO NOT READ: Don’t know  98 = DO NOT READ: Refused  99 = DO NOT READ: Not applicable |
| D01D. Women’s responsibilities take more time than men’s responsibilities do. | 0 = Fully disagree  1 = Partly disagree  2 = Partly agree  3 = Fully agree  97 = DO NOT READ: Don’t know  98 = DO NOT READ: Refused  99 = DO NOT READ: Not applicable |

|  |  | **(D03 A-F: USE SHOWCARD #2 - Optional)** |
| --- | --- | --- |
| **D02. INTERVIEWER, READ ALOUD:** Now I am going to ask you about the specific activities on which you spend time.  **NOTE TO INTERVIEWER: When examples are given, they should be read exactly as written and should NOT be skipped.** | **D02**. During the last 7 days, did you spend any time on [ACTIVITY], even if for one hour?  1 = Yes  2 = No  GOTO THE NEXT ACTIVITY UNTIL THIS QUESTION IS ASKED FOR ALL LISTED ACTIVITIES | **D03. INTERVIEWER, READ ALOUD:** Now I am going to ask you about how much influence you have in decisions about your time. I am going to list the same activities that we just discussed. For each activity, please rate **THE AMOUNT OF INFLUENCE YOU HAD OVER TIME YOU SPENT ON THE ACTIVITY DURINGTHE LAST SEVEN DAYS.** Please indicate whether you had **[show response card and point to the related response option on the card] no influence, some influence, or a lot of influence** regarding how you spent your time.  **D03a**. [IF D02A/B/C/D/E/F==1/Yes] During the last 7 days, did you have no influence, some influence, or a lot of influence in decisions about the amount of time you spent on [ACTIVITY]?  **D03b**. [IF D02A/B/C/D/E/F==2/No] During the last 7 days, did you have no influence, some influence, or a lot of influence in decisions about **not** spending time on [ACTIVITY]?  1 = No influence  2 = Some influence  3 = A lot of influence  97 = DO NOT READ: Don’t know  98 = DO NOT READ: Refused  99 = DO NOT READ: Not applicable |
| A. Household duties, such as cooking, cleaning, washing clothes, or collecting water or cooking fuel |  |  |
| B. Caring for household members, such as children or older family members |  |  |
| C. Going to the market to purchase essential items |  |  |
| D. Non-agricultural work activities, including: working for pay, in cash or in kind, for someone else; running or doing any kind of business or other activity to earn income; and helping in a family business |  |  |
| E. Agricultural production for sale, including: working on family farming or fishing activities to produce crop, livestock or fish products mainly for sale |  |  |
| F. Agricultural production for household consumption, including: working on family farming or fishing activities to produce crop, livestock or fish products mainly for household consumption |  |  |

| D_END. End of Section D date and time stamp: | DD | MM | YYYY | HH: | MM: | SS |
| --- | --- | --- | --- | --- | --- | --- |

**Section E: Participation and leadership in community organizations**

| E_START. Start of Section E date and time stamp: | DD | MM | YYYY | HH: | MM: | SS |
| --- | --- | --- | --- | --- | --- | --- |

**NOTE TO PROGRAMMER –DIRECTIONALITY OF HOW TO ASK QUESTIONS E01-E03- All items in E01 asked first (down the column), then E02 is asked and if the answer is Yes, then ask E03; if answer is anything other than Yes, ask E02 for the next item.**

|  | | **(E01 A-E: USE SHOWCARD #3 - Optional)** |  |  |
| --- | --- | --- | --- | --- |
| **INTERVIEWER, READ ALOUD:** Now, I will ask you about your participation in different types of organizations during the last 12 months. For each type of organization, please **indicate your LEVEL OF CONFIDENCE IN YOUR ABILITY TO PARTICIPATE in that type of organization [show response card and point to the related response option on the card] - whether you are not at all confident, somewhat confident, or very confident**. | | **E01.** Are you not at all confident, somewhat confident, or very confident in your ability to participate in [ORGANIZATION] if you wanted to?  Not at all confident 1  Somewhat confident 2  Very confident 3  DO NOT READ: Don’t know 97  DO NOT READ: Refused 98  DO NOT READ: Not applicable 99  >> NEXT ROW | **E02.** In the last 12 months, have you participated in [ORGANIZATION]?  Yes 1 >> Continue to E03  No 2 >> NEXT ROW  DO NOT READ: Don’t know …97 >> NEXT ROW  DO NOT READ: Refused …98 >> NEXT ROW  DO NOT READ: Not applicable…99>> NEXT ROW | **INTERVIEWER, READ ALOUD IF RESPONDENT PARTICIPATED IN AT LEAST ONE ORGANIZATION:** Now I will ask you about your leadership in the organiza-tion(s) in which you said you participated in the last 12 months.  **E03.** In the last 12 months, have you acted in a leadership position in [ORGANIZATION]?  Yes 1  No 2  DO NOT READ: Don’t know 97  DO NOT READ: Refused 98  DO NOT READ: Not applicable 99  >> NEXT ROW |
| **A** | Government councils or agencies |  |  |  |
| **B** | Groups that provide local services, such as resource user groups, community health and education service groups, or mutual aid groups |  |  |  |
| **C** | Formal or informal savings or credit groups, such as microfinance groups, village savings and loan associations, or merry-go-round savings schemes |  |  |  |
| **D** | Groups related to livelihood activities, such as agricultural groups, cooperatives, and craft or trade associations |  |  |  |
| **E** | Other groups, such as religious groups, women’s groups, or sports groups |  |  |  |

| **(USE SHOWCARD #1 - Optional)** | | |
| --- | --- | --- |
| **E04**. **INTERVIEWER, READ ALOUD:** Next I will read some statements about how women interact with the community. Please listen to each statement and **indicate [show response card and point to the related response option on the card] - whether you fully disagree, partly disagree, partly agree, or fully agree** with each statement: (items reduced for Malawi only; Bangladesh and Nepal used the full set A-I in E04 and E05) | | |
| **A** | Women can really understand what is going on with your community | Fully disagree 0  Partly disagree 1  Partly agree 2  Fully agree 3  DO NOT READ: Don’t know 97  DO NOT READ: Refused 98  DO NOT READ: Not applicable 99 |
| **B** | *Women have a pretty good understanding of the important issues that face your community* |  |
| **C** | Women have the ability to participate effectively in community activities |  |
| **D** | Women have the ability to participate effectively in decision-making |  |
| **E** | *Women’s opinion important because could someday make a difference in your community* |  |
| **F** | *There are plenty of ways for women to have a say in what your community does* |  |
| **G** | It is important to women that women actively participate in local women's issues |  |
| **H** | Most community leaders would listen to women |  |
| ***I*** | *It is important to women to participate in local activities* |  |
| **E05**. **INTERVIEWER, READ ALOUD:** Next I will read some statements about how men interact with the community. Please listen to each statement and **indicate [show response card and point to the related response option on the card] - whether you fully disagree, partly disagree, partly agree, or fully agree** with each statement: | | |
| **A** | Men can really understand what is going on with your community | Fully disagree 0  Partly disagree 1  Partly agree 2  Fully agree 3  DO NOT READ: Don’t know 97  DO NOT READ: Refused 98  DO NOT READ: Not applicable 99 |
| **B** | *Men have a pretty good understanding of the important issues that face your community* |  |
| **C** | Men have the ability to participate effectively in community activities |  |
| **D** | Men have the ability to participate effectively in decision-making |  |
| **E** | *Men’s opinion is important because it could someday make a difference in your community* |  |
| **F** | *There are plenty of ways for men to have a say in what your community does* |  |
| **G** | It is important to men that men actively participate in local men's issues |  |
| **H** | Most community leaders would listen to men |  |
| **I** | *It is important to men to participate in local activities* |  |

| E_END. End of Section E date and time stamp: | DD | MM | YYYY | HH: | MM: | SS |
| --- | --- | --- | --- | --- | --- | --- |

**Section F: Life transitions and awareness of rights**

| F_START. Start of Section F date and time stamp: | DD | MM | YYYY | HH: | MM: | SS |
| --- | --- | --- | --- | --- | --- | --- |

| **(USE SHOWCARD #1- Optional)** | | |
| --- | --- | --- |
| **F01. INTERVIEWER, READ ALOUD:** Now I’m going to read several statements of **PERCEPTIONS OF** **WOMEN’S RIGHTS**. Please listen to each statement and **indicate [show response card and point to the related response option on the card] - whether you Fully disagree, partly disagree, partly agree, or fully agree** with each statement. | | |
| **A** | Every woman should be free to choose whether to complete secondary school. | Fully disagree 0  Partly disagree 1  Partly agree..............................2  Fully agree 3  DO NOT READ: Don’t know 97  DO NOT READ: Refused 98  DO NOT READ: Not applicable 99 |
| **B** | Every woman should be free to choose whether to work for pay. |  |
| **C** | Every woman should be free to choose to prioritize her work for pay over domestic duties. |  |
| **D** | Every woman should be free to choose what to do with any money that she earns. |  |
| **E** | Every woman should be free to choose to purchase land, a house, or other valuable goods. |  |
| **F** | Every woman should be free to choose when to get married. |  |
| **G** | Every woman should be free to choose to divorce or end her marriage. |  |
| **H** | Every woman should be free to choose whether and when to have children. |  |
| **I** | Every woman should be free to choose not to have any more children. |  |

| F_END. End of Section F date and time stamp: | DD | MM | YYYY | HH: | MM: | SS |
| --- | --- | --- | --- | --- | --- | --- |

**Section G: Access to services and ownership of resources**

| G_START. Start of Section G date and time stamp: | DD | MM | YYYY | HH: | MM: | SS |
| --- | --- | --- | --- | --- | --- | --- |

| **INTERVIEWER, READ ALOUD:** Now, I am going to ask you whether you own or have access to certain things. | | |
| --- | --- | --- |
| **G01** | Do you have a government-issued identification card, such as a driver’s license, passport, citizenship card?  Examples in Nepal: citizenship certificate | Yes 1  No 2  DO NOT READ: Don’t know .97  DO NOT READ: Refused 98  DO NOT READ: Not applicable……………99 |
| **G02** | Do you own or have access to a mobile phone? | Yes, own 1  Yes, do not own but have access to…….....2  No, do not own or have access to 3  DO NOT READ: Don’t know .97  DO NOT READ: Refused 98  DO NOT READ: Not applicable 99 |
| **G03** | In the past 12 months, how often did you have access to each of the following services when you needed to? | |
| **A** | A person or place that you trust to take care of a dependent family member (who relies on you for care) for more than an hour | Never 1  Sometimes 2  Always 3  Did not need in last 12 months...................95  DO NOT READ: Don’t know .97  DO NOT READ: Refused 98  DO NOT READ: Not applicable 99 |
| **B** | Health care for yourself |  |
| **C** | Transportation that you can take to places outside your immediate community |  |
| **G04** | In the past 12 months, have you owned or had any of the following, either by yourself or jointly with someone else? | **(USE SHOWCARD #4 - Optional)** |
| **A** | A mobile money account such as Airtel Mobile Money or TNM Mpamba (Bangladesh “such as Bikash, Nagad, Roket, Upay, etc” | Yes, alone 1  Yes, jointly 2  Yes, both alone and jointly 3  No 4  DO NOT READ: Don’t know .97  DO NOT READ: Refused 98  DO NOT READ: Not applicable 99 |
| **B** | A bank account such as a savings account, current/checking account, or fixed account |  |
| **C** | A bank card or ATM card, (Not applicable in Malawi; Country teams to decide applicability of examples) |  |
| **D** | A credit card |  |
| **G05** | In the past 12 months, have you ever: | |
| **A** | Used any mobile money account, either yours or anyone else’s, to make a payment, buy something, or send money to someone? | Yes 1  No 2  DO NOT READ: Don’t know .97  DO NOT READ: Refused 98  DO NOT READ: Not applicable ……..99 |
| **B** | Deposited or received money into or withdrawn money from any bank account, either yours or anyone else’s? |  |
| **C** | Used any bank card, or ATM card, either yours or anyone else’s, to make a purchase or pay a bill such as a utility bill? (Not applicable in Malawi; Country teams to decide applicability of examples) |  |
| **D** | Used any credit card, either yours or anyone else’s, to make a purchase or pay a bill such as a utility bill? |  |

| **G06** | Could you take a loan from any of the following: | |
| --- | --- | --- |
| **A** | Bank or formal financial institution | Yes 1  No 2  DO NOT READ: Don’t know .97  DO NOT READ: Refused 98  DO NOT READ: Not applicable 99 |
| **B** | Cooperative |  |
| **C** | Group based micro-finance |  |
| **D** | Informal credit / savings groups, such as a village savings and loan group, merry-go-rounds, tontines, rosca, tanda, or funeral societies |  |
| **E** | Other NGO program |  |
| **F** | Friends or relatives |  |
| **G** | Other informal lender, such as a shopkeeper or moneylender |  |

| G_END. End of Section G date and time stamp: | DD | MM | YYYY | HH: | MM: | SS |
| --- | --- | --- | --- | --- | --- | --- |

**Section H: Property ownership**

| H_START. Start of Section H date and time stamp: | DD | MM | YYYY | HH: | MM: | SS |
| --- | --- | --- | --- | --- | --- | --- |

|  | **INTERVIEWER, READ ALOUD:** Now I am going to ask you about your ownership or right to use land and dwellings |  |
| --- | --- | --- |
| **H01** | Do you own or hold use rights to *any parcels of land*, either alone or jointly with someone else? **(USE SHOWCARD #4 - Optional)** | Yes, alone 1  Yes, jointly 2  Yes, both alone and jointly 3  No 4>>H08  DO NOT READ: Don’t know .97 >>H08  DO NOT READ: Refused 98 >>H08  DO NOT READ: Not applicable 99 >>H08 |
| **H02** | Do you own or hold use rights to agricultural land, non-agricultural land, or both? | Agricultural land only 1  Non-agricultural land only 2  Both agricultural and non-agricultural land 3  DO NOT READ: Don’t know .97  DO NOT READ: Refused 98  DO NOT READ: Not applicable .99 |
| **H03** | Do you have the right, either alone or jointly with someone else, to sell *any parcels of land* you own, or hold use rights to? **(USE SHOWCARD #4 - Optional)** | Yes, alone 1  Yes, jointly 2  Yes, both alone and jointly 3  No 4  DO NOT READ: Don’t know .97  DO NOT READ: Refused 98  DO NOT READ: Not applicable 99 |
| **H04** | Do you have the right, either alone or jointly with someone else, to give any parcels of land you own, or hold use rights to, by oral or written will, to other persons after your death? **(USE SHOWCARD #4 - Optional)** | Yes, alone 1  Yes, jointly 2  Yes, both alone and jointly 3  No 4  DO NOT READ: Don’t know .97  DO NOT READ: Refused 98  DO NOT READ: Not applicable 99 |
| **H05** | Is there a document for any parcels of land you own, or hold use rights to that is issued by or registered at the Land Registry/ Cadastral Agency, such as a title deed, certificate of ownership, certificate of hereditary acquisition, lease or rental contract??  **NOTE TO RESPONDENT:**  *Examples in Malawi: The document can be application for customary estate, offer of customary estate, acceptance of offer of customary estate, certificate of customary estate, freehold title, land certificate, certificate of official research, rental contract, lease contract..*  *Examples in Bangladesh: The document can be Barga contract, non-agricultural Khas land lease form, endorsement of certificate of registration, lease, occupation, verifying the current Khatian (record of rights).*  *Examples in Nepal: The document can be XXX.* | Yes 1  No 2 >>H07  DO NOT READ: Don’t know .97 >>H07  DO NOT READ: Refused 98 >>H07  DO NOT READ: Not applicable 99 >>H07 |
| **H05a** | What type of documents are there for parcels of land you own, or hold use rights to? [SELECT ALL THAT APPLY] | TITLE DEED.............. …………………………...1  (Bangladesh: REGISTERED DEED)  CERTIFICATE OF CUSTOMARY OWNERSHIP…….2  CERTIFICATE OF OCCUPANCY........ ………..3  CERTIFICATE OF HEREDITARY ……..….……4  ACQUISITION LISTED IN REGISTRY...............5  SURVEY PLAN............ ............... ......................6  RENTAL CONTRACT, REGISTERED...............7  LEASE, REGISTERED................... ...................8  (Bangladesh: Cropping deed, mortgaged deed)  OTHER (SPECIFY)...... ...................................96 |
| **H06** | Is your name listed on any of these documents as an owner or use right holder, either alone or jointly with someone else? **(USE SHOWCARD #4 - Optional)** | Yes, alone 1  Yes, jointly 2  Yes, both alone and jointly 3  No 4  DO NOT READ: Don’t know .97  DO NOT READ: Refused 98  DO NOT READ: Not applicable 99 |
| **H07** | How likely are you to involuntarily lose ownership or use rights to any land you own, or hold use rights to in the next 5 years?  **READ RESPONSE OPTIONS** | Not all likely……………………………..………..1  Slightly likely……………………………..……….2  Moderately likely………………………..………..3  Very likely………………………….…….………..4  Extremely likely……………………………….….5  DO NOT READ: Don’t know. .97  DO NOT READ: Refused 98  DO NOT READ: Not applicable 99 |
| A dwelling is defined as a room or suite of rooms in a permanent building or structurally separated part of a building (e.g., a unit in an apartment building), that is used for private habitation by one or more households.  Please ask questions H08 – H19 if ANY of the following conditions are met by the study context (possibly by country or specific region within a country):  □ Land is owned by government and individuals can own and/or rent dwelling units built on specific land areas.  □ Individuals can own and/or rent dwelling units, such as an apartment or a condo, without land ownership.  □ Individuals can own and/or rent land and dwelling individually. (For Country teams to decide if the following questions are applicable to the local context- Should NOT be visible in CAPI version of questionnaire) | | |
| **H08** | Do you own or hold use rights to the dwelling in which you live, either alone or jointly with someone else? **(USE SHOWCARD #4 - Optional)** | Yes, alone 1  Yes, jointly 2  Yes, both alone and jointly 3  No 4  DO NOT READ: Don’t know .97  DO NOT READ: Refused 98  DO NOT READ: Not applicable 99 |
| **H09** | Do you have the right, either alone or jointly with someone else, to sell the dwelling in which you live?  **(USE SHOWCARD #4 - Optional)**  CAPI PROGRAMMING NOTE: THIS QUESTION IS ENABLED ONLY IF H08==1, or 2, or 3 | Yes, alone 1  Yes, jointly 2  Yes, both alone and jointly 3  No 4  DO NOT READ: Don’t know .97  DO NOT READ: Refused 98  DO NOT READ: Not applicable 99 |
| **H10** | Do you have the right, either alone or jointly with someone else, to give your residential dwelling, by oral or written will, to other persons after your death? **(USE SHOWCARD #4 - Optional)**  CAPI PROGRAMMING NOTE: THIS QUESTION IS ENABLED ONLY IF H08==1, or 2, or 3 | Yes, alone 1  Yes, jointly 2  Yes, both alone and jointly 3  No 4  DO NOT READ: Don’t know .97  DO NOT READ: Refused 98  DO NOT READ: Not applicable 99 |
| **H11** | Is there a document for the residential dwelling you own, or hold use rights to that is issued by or registered at a government agency, such as a title deed, certificate of ownership, certificate of hereditary acquisition, lease or rental contract?  *Examples in Malawi: The document can be application for customary estate, offer of customary estate, acceptance of offer of customary estate, certificate of customary estate, freehold title, land certificate, certificate of official research, rental contract, lease contract*  *Examples in Bangladesh: The document can be Barga contract, non-agricultural Khas land lease form, endorsement of certificate of registration, lease, occupation, verifying the current Khatian (record of rights).*  *Examples in Nepal: The document can be XXX*  **CAPI PROGRAMMING NOTE**: THIS QUESTION IS ENABLED ONLY IF H08==1, or 2, or 3 | Yes 1  No 2 >>H14  DO NOT READ: Don’t know .97 >>H14  DO NOT READ: Refused 98 >>H14  DO NOT READ: Not applicable 99 >>H14 |
| **H11a** | What type of documents are there for the residential dwelling you own, or hold use rights to? [SELECT ALL THAT APPLY] (Country teams should confirm whether there are revisions needed to this list.) | TITLE DEED.......................................................1  CERTIFICATE OF CUSTOMARY OWNERSHIP2  CERTIFICATE OF OCCUPANCY…..….............3  CERTIFICATE OF HEREDITARY ….………..…4  ACQUISITION LISTED IN REGISTRY ..............5  SURVEY PLAN..................................................6  RENTAL CONTRACT, REGISTERED...............7  LEASE, REGISTERED.......................................8  OTHER (SPECIFY)..........................................96 |
| **H12** | Is your name listed on any of these documents as an owner or use right holder, either alone or jointly with someone else?  **NOTE TO RESPONDENT:** The document can be [TO BE COMPLETED FOR COUNTRY-SPECIFIC CONTEXT]  **(USE SHOWCARD #4 - Optional)**  **CAPI PROGRAMMING NOTE**: THIS QUESTION IS ENABLED ONLY IF H11==1 | Yes, alone 1  Yes, jointly 2  Yes, both alone and jointly 3  No 4  DO NOT READ: Don’t know .97  DO NOT READ: Refused 98  DO NOT READ: Not applicable 99 |
| **H13** | How likely are you to involuntarily lose ownership to the residential dwelling you own, or hold use rights to in the next 5 years?  **READ RESPONSE OPTIONS**  **CAPI PROGRAMMING NOTE**: THIS QUESTION IS ENABLED ONLY IF H08==1, or 2, or 3 | Not all likely………………………………………..1  Slightly likely……………………………………….2  Moderately likely…………………………………..3  Very likely………………………………..….……..4  Extremely likely……………….…………….…….5  DO NOT READ: Don’t know .97  DO NOT READ: Refused 98  DO NOT READ: Not applicable 99 |

| **H14** | Do you own or hold use rights to any other dwelling, either alone or jointly with someone else? **(USE SHOWCARD #4 - Optional)** | Yes, alone 1  Yes, jointly 2  Yes, both alone and jointly 3  No 4  DO NOT READ: Don’t know .97  DO NOT READ: Refused 98  DO NOT READ: Not applicable 99 |
| --- | --- | --- |
| **H15** | Do you have the right, either alone or jointly with someone else, to sell any other dwellings you own?  **(USE SHOWCARD #4 - Optional)**  CAPI PROGRAMMING NOTE: THIS QUESTION IS ENABLED ONLY IF H14==1, or 2, or 3 | Yes, alone 1  Yes, jointly 2  Yes, both alone and jointly 3  No 4  DO NOT READ: Don’t know .97  DO NOT READ: Refused 98  DO NOT READ: Not applicable 99 |
| **H16** | Do you have the right, either alone or jointly with someone else, to give any other dwellings you own, by oral or written will, to other persons after your death? **(USE SHOWCARD #4 - Optional)**  CAPI PROGRAMMING NOTE: THIS QUESTION IS ENABLED ONLY IF H14==1, or 2, or 3 | Yes, alone 1  Yes, jointly 2  Yes, both alone and jointly 3  No 4  DO NOT READ: Don’t know .97  DO NOT READ: Refused 98  DO NOT READ: Not applicable 99 |
| **H17** | Is there a document for any other dwellings you own, or hold use rights to that is issued by or registered at a government agency, such as a title deed, certificate of ownership, certificate of hereditary acquisition, lease or rental contract?  *Examples in Malawi: The document can be application for customary estate, offer of customary estate, acceptance of offer of customary estate, certificate of customary estate, freehold title, land certificate, certificate of official research, rental contract, lease contract*  *Examples in Bangladesh: The document can be Barga contract, non-agricultural Khas land lease form, endorsement of certificate of registration, lease, occupation, verifying the current Khatian (record of rights).*  *Examples in Nepal: The document can be XXX*  **CAPI PROGRAMMING NOTE**: THIS QUESTION IS ENABLED ONLY IF H14==1, or 2, or 3 | Yes 1  No 2 >>H14  DO NOT READ: Don’t know .97 >>H14  DO NOT READ: Refused 98 >>H14  DO NOT READ: Not applicable 99 >>H14 |
| **H17a** | What type of documents are there for any other dwellings you own, or hold use rights to? [SELECT ALL THAT APPLY]  (Country teams should confirm whether there are revisions needed to this list.) | TITLE DEED......................................................1  CERTIFICATE OF CUSTOMARY OWNERSHIP2  CERTIFICATE OF OCCUPANCY…..............3  CERTIFICATE OF HEREDITARY …………..…4  ACQUISITION LISTED IN REGISTRY .............5  SURVEY PLAN..................................................6  RENTAL CONTRACT, REGISTERED...............7  LEASE, REGISTERED......................................8  OTHER (SPECIFY).........................................96 |
| **H18** | Is your name listed on any of these documents as an owner or use right holder, either alone or jointly with someone else?  **NOTE TO RESPONDENT:** The document can be [TO BE COMPLETED FOR COUNTRY-SPECIFIC CONTEXT]  **(USE SHOWCARD #4 - Optional)**  **CAPI PROGRAMMING NOTE**: THIS QUESTION IS ENABLED ONLY IF H17==1 | Yes, alone 1  Yes, jointly 2  Yes, both alone and jointly 3  No 4  DO NOT READ: Don’t know .97  DO NOT READ: Refused 98  DO NOT READ: Not applicable 99 |
| **H19** | How likely are you to involuntarily lose ownership to any of the other dwellings you own, or hold use rights to in the next 5 years?  **READ RESPONSE OPTIONS**  **CAPI PROGRAMMING NOTE**: THIS QUESTION IS ENABLED ONLY IF H14==1, or 2, or 3 | Not all likely………………………………………..1  Slightly likely……………………………………….2  Moderately likely…………………………………..3  Very likely………………………………..….……..4  Extremely likely……………….…………….…….5  DO NOT READ: Don’t know .97  DO NOT READ: Refused 98  DO NOT READ: Not applicable 99 |

| H_END. End of Section H date and time stamp: | DD | MM | YYYY | HH: | MM: | SS |
| --- | --- | --- | --- | --- | --- | --- |

**Section I: Decision-making/Control over income**

| I_START. Start of Section I date and time stamp: | DD | MM | YYYY | HH: | MM: | SS |
| --- | --- | --- | --- | --- | --- | --- |

| **I01. INTERVIEWER, READ ALOUD:** Now I am going to ask you about how much influence you have in decisions and control over income. For each activity, please rate **THE AMOUNT OF INFLUENCE YOU HAD OVER DECISIONS AND CONTROL OVER INCOME** Please indicate whether you had **[show response card and point to the related response option on the card] no influence, some influence, or a lot of influence** regarding your input in the following decisions and control over income. **(USE SHOWCARD #2- Optional)** | | |
| --- | --- | --- |
|  | How much influence do you have regarding control over any source of income, **no influence, some influence, or a lot of influence**? | No influence 1  Some influence 2  A lot of influence 3  DO NOT READ: Don’t know .97  DO NOT READ: Refused 98  DO NOT READ: Not applicable 99 |
| **I02** | Now, please tell me whether you have **no influence, some influence, or a lot of influence** in decisions about the following items: | **(I02A-C: USE SHOWCARD #2- Optional)** |
| **A** | Large household purchases | No influence 1  Some influence 2  A lot of influence 3  DO NOT READ: Don’t know .97  DO NOT READ: Refused 98  DO NOT READ: Not applicable 99 |
| **B** | Minor household purchases |  |
| **C** | Own healthcare |  |

| I_END. End of Section I date and time stamp: | DD | MM | YYYY | HH: | MM: | SS |
| --- | --- | --- | --- | --- | --- | --- |

**Section J: Access and use of ICT**

| J_START. Start of Section J date and time stamp: | DD | MM | YYYY | HH: | MM: | SS |
| --- | --- | --- | --- | --- | --- | --- |

| **(USE SHOWCARD #5- Required)** | | |
| --- | --- | --- |
| **J01. INTERVIEWER, READ ALOUD:** Next, I am going to ask you **how often** you use **different types of technology.** For each type of technology, please indicate  **[show response card and point to the related response option on the card] - whether you use** **the** technology **daily, weekly, monthly, less than monthly, or never**. | | |
| **A** | Radio | Daily 1  Weekly 2  Monthly 3  Less than monthly 4  Never 5  DO NOT READ: Don’t know .97  DO NOT READ: Refused 98 |
| **B** | Television |  |
| **C** | Landline telephone |  |
| **D** | Mobile phone |  |
| **E** | Computer, laptop, or computer tablet, such as an iPad or notebook computer |  |
| **F** | Internet |  |

| J_END. End of Section J date and time stamp: | DD | MM | YYYY | HH: | MM: | SS |
| --- | --- | --- | --- | --- | --- | --- |

**Section K: Validation**

| K_START. Start of Section K date and time stamp: | DD | MM | YYYY | HH: | MM: | SS |
| --- | --- | --- | --- | --- | --- | --- |

| **K01** | **INTERVIEWER, READ ALOUD:** Now I would like to ask you some questions about your health, your well-being, and your opinion on several matters.  Taking all things together, would you say you are (*read out all response options and code one answer*):  **(USE SHOWCARD #6 - Optional)** | | | | | | | | Very happy 1  Somewhat happy 2  Not very happy 3  Not at all happy 4 | | | |
| --- | --- | --- | --- | --- | --- | --- | --- | --- | --- | --- | --- | --- |
| **K02** | All in all, how would you describe your state of health these days? Would you say your state of health is… (*read out all response options and code one answer*):  **(USE SHOWCARD #7- Optional)** | | | | | | | | Very good 1  Good 2  Fair 3  Poor 4  Very poor 5 | | | |
| **K03** | Some people feel they have completely free choice and control over their lives, while other people feel that what they do has no real effect on what happens to them. Please use this scale **[****SHOWCARD #8 - Required**] where 1 means "no choice at all" and 4 means "a great deal of choice" to indicate how much freedom of choice and control you feel you have over the way your life turns out (*code one number*): | | | | | | | | No choice at all 1  A little choice 2  Some choice 3  A great deal of choice .4  DO NOT READ: Don’t know .97  DO NOT READ: Refused 98 | | | |
| **K04** | All things considered, how satisfied are you with your life as a whole these days? Using this card [**SHOWCARD #9- Required]** on which 1 means you are “completely dissatisfied” and 4 means you are “completely satisfied” where would you put your satisfaction with your life as a whole? (*Code one number*): | | | | | | | | Completely dissatisfied 1  Partly dissatisfied 2  Partly satisfied 3  Completely satisfied… 4  DO NOT READ: Don’t know .97  DO NOT READ: Refused 98 | | | |
| **K05** | How satisfied are you with the financial situation of your household? Please use this card again [**SHOWCARD #9- Required]** to help with your answer (*code one number*): | | | | | | | | Completely dissatisfied 1  Partly dissatisfied 2  Partly satisfied 3  Completely satisfied… .4  DO NOT READ: Don’t know .97  DO NOT READ: Refused 98 | | | |
| **K06** | In the last 12 months, how often have you gone without enough food to eat: often, sometimes, rarely, or never?  **[USE SHOWCARD #10- Required]** | | | | | | | | Often 1  Sometimes 2  Rarely 3  Never 4  DO NOT READ: Don’t know .97  DO NOT READ: Refused 98  DO NOT READ: Not applicable…..….99 | | | |
| **K07** | In the last 12 months, how often have you felt unsafe from crime in your home: often, sometimes, rarely, or never? **[USE SHOWCARD #11- Optional]** | | | | | | | | Often 1  Sometimes 2  Rarely 3  Never 4  DO NOT READ: Don’t know .97  DO NOT READ: Refused 98  DO NOT READ: Not applicable..…….99 | | | |
| **INTERVIEWER, READ ALOUD:** Now I'd like you to tell me your views on various issues. How would you place your views on this scale? 1 means you agree completely with the statement on the left; 4 means you agree completely with the statement on the right; and if your views fall somewhere in between, you can choose any number in between. (*Code one number for each issue*) **ENUMERATOR NOTE: READ OUT LOUD THE STATEMENTS AT BOTH ENDS OF THE SCALE**: | | | | | | | | | | | | |
| **K08** | **INTERVIEWER, READ ALOUD:** On a scale of 1 to 4, where 1 means you agree completely that incomes should be more equal and 4 means you agree completely that there should be greater incentives for individual effort, how would you place your views? If your views fall somewhere in between, you can choose a number in between 1 and 4 | | | | | | | | | | | |
|  | Incomes should be more equal |  |  | There should be greater incentives for individual effort |  |  |  |  | | |  |  |
|  | 1 | 2 | 3 | 4 |  |  |  |  | | |  |  |
| **K09** | **INTERVIEWER, READ ALOUD:** On a scale of 1 to 4, where 1 means you agree completely that the government should take more responsibility to ensure that everyone is provided for and 4 means you agree completely that people should take more responsibility to provide for themselves, how would you place your views? If your views fall somewhere in between, you can choose a number in between 1 and 4. | | | | | | | | | | | |
|  | Government should take more responsibility to ensure that everyone is provided for |  |  | People should take more responsibility to provide for themselves |  |  |  |  | | |  |  |
|  | 1 | 2 | 3 | 4 |  |  |  |  | | |  |  |
| **INTERVIEWER, READ ALOUD:** Please tell me for each of the following actions whether you think it can always be justified, sometimes justifiable, rarely justifiable, or never be justified, using this card **[USE SHOWCARD #12 - Required]**. (*Read out and code one answer for each statement*): | | | | | | | | | | | | |
| **K10** | Divorce | | | | | | | | | Always justifiable 1  Sometimes justifiable 2  Rarely justifiable 3  Never justifiable .4  DO NOT READ: Don’t know .97  DO NOT READ: Refused 98 | | |
| **K11** | For a man to beat his wife | | | | | | | | | Always justifiable 1  Sometimes justifiable 2  Rarely justifiable 3  Never justifiable .4  DO NOT READ: Don’t know .97  DO NOT READ: Refused 98 | | |

| **(USE SHOWCARD #5 - Required)** | | |
| --- | --- | --- |
| **K12** | **INTERVIEWER, READ ALOUD:** People learn what is going on in this country and the world from various sources. For each of the following sources, please indicate whether you use it to obtain information daily, weekly, monthly, less than monthly or never (*read out and code one answer for each*): | |
| **A** | Daily newspaper | Daily 1  Weekly 2  Monthly 3  Less than monthly 4  Never 5  DO NOT READ: Don’t know .97  DO NOT READ: Refused 98 |
| **B** | TV news |  |
| **C** | Radio news |  |
| **D** | Mobile phone |  |
| **E** | Email |  |
| **F** | Internet |  |
| **G** | Social media (Facebook, Twitter, etc.) |  |
| **H** | Talk with friends or colleagues |  |

| **K13** | Does anyone in your household currently have any [ITEM]? | |
| --- | --- | --- |
| **A** | Mat/matela or bed | No 0  Yes 1  DO NOT READ: Don’t know .97  DO NOT READ: Refused 98 |
| **B** | Bicycle |  |
| **C** | Motorcycle/scooter |  |
| **D** | Car/Truck |  |
| **E** | Mobile phone |  |
| **F** | Radio |  |
| **G** | Television |  |
| **H** | Clothes washing machine |  |
| **I** | Couch/ Sofa |  |

| **K14** | **INTERVIEWER, READ ALOUD:** Imagine that you have an emergency and you need to pay [insert 1/20 of GNI per capita in local currency]. How possible is it that you could come up with [*insert 1/20 of GNI per capita in local currency*] within the next month? (Country teams to update with relevant value.) | Very possible 1  Somewhat possible 2  Not very possible 3  Not at all possible 4  DO NOT READ: Don’t know .97  DO NOT READ: Refused 98  DO NOT READ: Not applicable 99 |
| --- | --- | --- |
| **K15** | In the last 7 days, did you work as an unpaid apprentice even if just for one hour? | Yes 1  No 2  DO NOT READ: Don’t know .97  DO NOT READ: Refused 98  DO NOT READ: Not applicable……………….99 |
| **K16** | In the last 7 days, did you work as an employee for a wage, salary, commission or any payment in kind; including doing a paid apprenticeship, domestic work or paid farm work even if just for one hour? |  |
| **K17** | In the last 7 days, did you run a non-farm business of any size for yourself or the household, even if just for one hour? |  |
| **K18** | In the last 7 days, did you help in any kind of non-farm business run by this household, even if just for one hour? |  |
| **K19** | In the last 7 days, did you work on household agricultural activities (including farming, raising livestock or fishing, whether for sale or for household food) even if just for one hour? |  |

| **Topic** | | **K20** How much input do you have—no input or input into few decisions, input into some decisions, or input into most or all decisions--in making decisions about [TOPIC]? | **K21** To what extent—not at all, to a small extent, to a medium extent, or to a high extent--do you feel you can make your own personal decisions regarding [TOPIC] if you want(ed) to? |
| --- | --- | --- | --- |
| **A** | Income you earn | No input or input into few decisions 1  Input into some decisions 2  Input into most or all decisions 3  DO NOT READ: Don’t know .97  DO NOT READ: Refused 98  DO NOT READ: Not applicable…………….99 | Not at all 1  Small extent 2  Medium extent 3  High extent 4  DO NOT READ: Don’t know .97  DO NOT READ: Refused 98  DO NOT READ: Not applicable 99 |
| **B** | How household income is spent |  |  |
| **C** | Major household expenses |  |  |

| K_END. End of Section J date and time stamp: | DD | MM | YYYY | HH: | MM: | SS |
| --- | --- | --- | --- | --- | --- | --- |

**Section J: Sexual Harassment**

| L_START. Start of Section J date and time stamp: | DD | MM | YYYY | HH: | MM: | SS |
| --- | --- | --- | --- | --- | --- | --- |

| **L00** | *DO NOT READ ALOUD: INTERVIEWER: PLEASE MAKE SURE THERE IS NO PERSON 5 YEARS AND OVER WHO COULD OVERHEAR THE INTERVIEW. Interviewer, are you in a place where the questions and responses will not be overheard by anyone older than five years old?* | |
| --- | --- | --- |
|  | ***INTERVIEWER READ ALOUD: Now, I will ask you some questions about experiences women in your community may have. In your opinion, is it “never acceptable,” “sometimes acceptable,” or “usually acceptable.” for the following to happen*** | |
| **L01** | A man treats a woman as “lesser” because she is a woman, for example, speaks badly, interrupts, or ignores her? | Never acceptable 1  Sometimes acceptable 2  Usually acceptable 3  DO NOT READ: Don’t know 97  DO NOT READ: Refused 98  DO NOT READ: Not applicable 99 |
| **L02** | A man prevents a woman from doing certain kinds of work, even if she wants to? | Never acceptable 1  Sometimes acceptable 2  Usually acceptable 3  DO NOT READ: Don’t know 97  DO NOT READ: Refused 98  DO NOT READ: Not applicable 99 |
| **L03** | A man spreads unwanted rumors about a woman’s sex life? | Never acceptable 1  Sometimes acceptable 2  Usually acceptable 3  DO NOT READ: Don’t know 97  DO NOT READ: Refused 98  DO NOT READ: Not applicable 99 |
| **L04** | A man tries to have a romantic or sexual relationship with a woman when she doesn’t want it? | Never acceptable 1  Sometimes acceptable 2  Usually acceptable 3  DO NOT READ: Don’t know 97  DO NOT READ: Refused 98  DO NOT READ: Not applicable 99 |
| **L05** | A man offers work-related benefits to a woman with the expectation of receiving sexual favors? | Never acceptable 1  Sometimes acceptable 2  Usually acceptable 3  DO NOT READ: Don’t know 97  DO NOT READ: Refused 98  DO NOT READ: Not applicable 99 |

| L_END. End of Section J date and time stamp: | DD | MM | YYYY | HH: | MM: | SS |
| --- | --- | --- | --- | --- | --- | --- |

**Section Z: Result of the Interview**

**INTERVIEWER READ OUT**: Thank you very much for your participation in this survey! I may try to contact you in future for another short interview. Before you go, I have a couple of questions to help in case I need to contact you in future.

| Z01. Do you have an alternate phone number? It will be helpful for us to contact you through the alternate number in case you may be unavailable on this number. (BANGLADESH ONLY) | Yes ............1 >> Z2  No ................2 |
| --- | --- |
| Z02. Please provide me your alternate phone number. (BANGLADESH ONLY) |  |
| INTERVIEWER CONFIRM THAT ALL QUESTIONS HAVE BEEN ANSWERED.  READ OUT: That's it for now. Thank you very much for answering all questions and helping us know more about your ideas and opinions. This information is important.  If you have any questions about the survey, you can call [COUNTRY-SPECIFIC PHONE NUMBER HERE] | |
| Z03. INTERVIEWER NOTES: PLEASE MAKE ANY NOTES HERE REGARDING YOUR IMPRESSIONS OF THE INTERVIEW, FOR EXAMPLE IF THE RESPONDENT WAS UNCOMFORTABLE ANSWERING A QUESTION, IF OTHER HOUSEHOLD MEMBERS WERE TRYING TO LISTEN TO THE INTERVIEW, ETC. | |
| Z04. Main language of enumerator (enter code) | |
| Z05. Main language of respondent (enter code) | |
| Z06. Outcome of Interview (enter code) | |

| **Code 1: (B02) Language**  **ADAPT TO LOCAL CONTEXT** | **OUTCOME OF VISIT** |
| --- | --- |
| English ………………………………...1  ChiChewa…………………………….. 2  ChiYao…………………….…………...3  ChiTimbuka………………………..…..4  Bangla………………………..…..…5  Other, specify…………………….…..96 | 01 = Successful/Complete  02 = Entire household absent outside the area for extended period of time  03 = Refused (in general)  04 = Refused to consent  05 = Postponed (RETURN FOR CALL-BACK)  06 = No household member at home or no competent respondent at home at time of visit (RETURN FOR CALL-BACK)  07 = Partial (RETURN FOR CALL-BACK)  96 = Other (specify) ________________ |

| END_TIME. End of survey date and time stamp: | DD | MM | YYYY | HH: | MM: | SS |
| --- | --- | --- | --- | --- | --- | --- |

**Initial exploratory factor analyses of WEMNS**

| **# of Factors** | **Range of Factor Loadings** | **RMSEA (90% CI)** | **SRMR** | **CFI** | **TLI** | **Range of Factor Correlations** |
| --- | --- | --- | --- | --- | --- | --- |
|  |  |  |  |  |  | **Resources** |
| 1 | 0.000-0.852 | 0.014 (0.013, 0.015) | 0.169 | 0.662 | 0.653 | N/A |
| 2 | 0.000-0.801 | 0.010 (0.009, 0.011) | 0.148 | 0.821 | 0.811 | 0.186 |
| 3 | 0.002-0.771 | 0.009 (0.007, 0.010) | 0.127 | 0.876 | 0.865 | -0.025 – 0.185 |
| 4 | 0.000--0.921 | 0.008 (0.006, 0.009) | 0.106 | 0.900 | 0.889 | -0.188 – 0.178 |
| 5 | 0.001-0.953 | 0.006 (0.005, 0.008) | 0.088 | 0.934 | 0.924 | -0.189 – 0.234 |
| 6 | 0.000-0.934 | 0.006 (0.004, 0.008) | 0.082 | 0.944 | 0.934 | -0.219 – 0.322 |
| 7 | 0.000-0.938 | 0.006 (0.003, 0.007) | 0.074 | 0.954 | 0.944 | -0.247 – 0.367 |
| 8 | 0.002-0.946 | 0.005 (0.002, 0.007) | 0.065 | 0.962 | 0.953 | -0.245 – 0.336 |
| Note: EFA models with 9-13 factors failed to converge. | | | | | | |

# **Expected relationships of WEMNS derived factor scores with external measures**

|  | **Self-report measures to assess convergent or discriminant construct validity** | | | | | | | | | | | |
| --- | --- | --- | --- | --- | --- | --- | --- | --- | --- | --- | --- | --- |
|  | **K01** | **K02** | **K03** | **K04** | **K05** | **K06** | **K10** | **K11** | **K12D** | **12F** | **K20** | **K21** |
| **WEMNS Dimensions (factor scores derived from final CFA model in pooled sample)** | **Happiness** | **Health** | **Extent of choice over one's life** | **Life satisfaction** | **Satisfaction with HH financial situation** | **Freq went w/o food last 12 months** | **Extent divorce justified** | **Extent wife beating justified** | **Freq of obtaining info from mobile phone** | **Freq of obtaining info from internet** | **Input into decisions about HH income, spending** | **Ability to make own decisions about HH income, spending** |
| **Use of financial services^1^** | + | + | + | + | + | - | + | - | + | + | + | + |
| **Access to credit^1^** | ? | + | + | ? | ? | - | + | - | + | + | + | + |
| **Decision-making and control over income^1^** | + | + | + | + | + | - | + | - | + | + | + | + |
| **Access to / use of ICT^2^** | ? | + | + | ? | + | - | + | - | + | + | + | + |
| **Endorsement of women’s freedom to choose her preferences in livelihoods** | ? | ? | + | ? | + | - | + | - | + | + | + | + |
| **Endorsement of women’s freedom to choose her preferences in family formation** | ? | ? | + | ? | + | - | + | - | + | + | + | + |
| **Endorsement of forms of sexual harassment against women** | ? | ? | - | ? | ? | + | - | + | - | - | - | - |
| **Awareness of gender inequalities in time allocated to paid, unpaid activities** | - | - | - | - | - | ? | + | - | + | + | + | + |
| **Ability to influence own time allocation** | + | + | + | + | + | - | + | - | + | + | + | + |
| **Confidence to participate in community organizations^3,4^** | + | + | + | + | + | - | + | - | + | + | + | + |
| **Participation, leadership in community organizations^3,4,5^** | ? | ? | + | ? | + | - | + | - | + | + | + | + |
| **Perceptions of women’s community engagement^3,4^** | + | + | + | + | + | - | + | - | + | + | + | + |
| **Perceptions of men’s community engagement** | ? | ? | ? | ? | ? | - | ? | ? | ? | ? | ? | ? |
| + denotes expected positive relationship (test of convergent validity) |  |  |  |  |  |  |  |  |  |  |  |  |
| - denotes expected negative relationship (test of discriminant validity) |  |  |  |  |  |  |  |  |  |  |  |  |
| ? Denotes nature of relationship unknown or potentially complex |  |  |  |  |  |  |  |  |  |  |  |  |
| ^1^ McGuire, J., Kaiser, C., & Bach-Mortensen, A. (2020). The impact of cash transfers on subjective well-being and mental health in low-and middle-income countries: A systematic review and meta-analysis. | | | | | | | | | |  |  |  |
| ^2^ Meherali, S., Rahim, K. A., Campbell, S., & Lassi, Z. S. (2021). Does digital literacy empower adolescent girls in low-and middle-income countries: a systematic review. Frontiers in Public Health, 9, 761394. | | | | | | | | | |  |  |  |
| ^3^ Conlin, S. E., Douglass, R. P., Moradi, B., & Ouch, S. (2021). Examining Feminist and Critical Consciousness Conceptualizations of Women’s Subjective Well-Being. The Counseling Psychologist, 49(3), 391–422. | | | | | | | | | | |  |  |
| ^4^ De Hoop, T., van Kempen, L., Linssen, R., & van Eerdewijk, A. (2014). Women's autonomy and subjective well-being: how gender norms shape the impact of self-help groups in Odisha, India. Feminist economics, 20(3), 103-135. | | | | | | | | | | | |  |
| ^5^ Relation with measures of subjective well-being may vary by normative context. |  |  |  |  |  |  |  |  |  |  |  |  |
